# Neurobiology of resilience in depression: immune and vascular insights from human and animal studies

**DOI:** 10.1111/ejn.14547

**Published:** 2019-09-13

**Authors:** Katarzyna A. Dudek, Laurence Dion‐Albert, Fernanda Neutzling Kaufmann, Ellen Tuck, Manon Lebel, Caroline Menard

**Affiliations:** ^1^ Department of Psychiatry and Neuroscience Faculty of Medicine and CERVO Brain Research Center Université Laval Quebec City QC Canada; ^2^ Smurfit Institute of Genetics Trinity College Dublin Ireland

**Keywords:** blood–brain barrier, cytokines, depression, immune, sex differences, stress, vascular

## Abstract

Major depressive disorder (MDD) is a chronic and recurrent psychiatric condition characterized by depressed mood, social isolation and anhedonia. It will affect 20% of individuals with considerable economic impacts. Unfortunately, 30–50% of depressed individuals are resistant to current antidepressant treatments. MDD is twice as prevalent in women and associated symptoms are different. Depression's main environmental risk factor is chronic stress, and women report higher levels of stress in daily life. However, not every stressed individual becomes depressed, highlighting the need to identify biological determinants of stress vulnerability but also resilience. Based on a reverse translational approach, rodent models of depression were developed to study the mechanisms underlying susceptibility vs resilience. Indeed, a subpopulation of animals can display coping mechanisms and a set of biological alterations leading to stress resilience. The aetiology of MDD is multifactorial and involves several physiological systems. Exacerbation of endocrine and immune responses from both innate and adaptive systems are observed in depressed individuals and mice exhibiting depression‐like behaviours. Increasing attention has been given to neurovascular health since higher prevalence of cardiovascular diseases is found in MDD patients and inflammatory conditions are associated with depression, treatment resistance and relapse. Here, we provide an overview of endocrine, immune and vascular changes associated with stress vulnerability vs. resilience in rodents and when available, in humans. Lack of treatment efficacy suggests that neuron‐centric treatments do not address important causal biological factors and better understanding of stress‐induced adaptations, including sex differences, could contribute to develop novel therapeutic strategies including personalized medicine approaches.

## Graphical abstract

Endocrine, immune and vascular aspects contributing to depression and resilience. Evidence from mouse and human studies highlighted sex‐specific roles of these three different, but associated, systems in major depressive disorder and resilience.

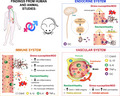

**Abbreviations**
ACTH: adrenocorticotropin hormone
ANS: autonomic nervous system
AQP4: aquaporin 4
BBB: blood–brain barrier
BDNF: brain‐derived neurotrophic factor
CCL2: chemokine ligand 2
CCR2: chemokine receptor 2
CLDN5: claudin‐5
CMS: chronic mild stress
CNS: central nervous system
CRF: corticotropin‐releasing factor
CRH: corticotropin‐releasing hormone
CRS: chronic restraint stress
CSDS: chronic social defeat stress
CSF: cerebrospinal fluid
CX3CR1: fractaline receptor 1
CXCR3: CXC chemokine receptor 3
DC: dendritic cells
ELS: early‐life stress
ERα/β: oestrogen receptor alpha/beta
GC: glucocorticoid
GFAP: glial fibrillary acidic protein
HAMD: Hamilton Depression Rating Scale
HPA: hypothalamic–pituitary–adrenal
Iba1: ionized calcium binding adaptor molecular 1
ICAM1: intercellular adhesion molecular 1
IFN‐γ: interferon‐gamma
IL‐1R1: interleukin‐1 receptor type 1
IL: interleukin
LC: locus coeruleus
LH: learned helplessness
LPS: lipopolysaccharide
Ly6C: lymphocyte Ag 6 high monocytes
MADRS: Montgomery and Asberg Depression Rating Scale
MDD: major depressive disorder
MRI: magnetic resonance imaging
NAc: nucleus accumbens
NE: norepinephrine
NK: natural killer cells
NLRP3: nucleotide‐binding domain and leucin‐rich repeat protein‐3
NVU: neurovascular unit
P‐gp: P‐glycoprotein transporters
PVN: paraventricular nucleus
RSD: repeated social defeat
RUR: relative uptake ratio
SES: socioeconomic status
SI: social interaction
TLR4: toll‐like receptor 4
TNF‐α: tumour necrosis factor‐alpha
VCAM‐1: vascular cell adhesion molecular 1
VEGF: vascular endothelial growth factor
ZO: zonula occludens

## 1 INTRODUCTION

Over 300 million people are suffering from depression worldwide (World Health Organisation, 2017) with an estimated one out of five individuals affected by the most prevalent form of depression, major depressive disorder (MDD), through their lifetime (Kessler, Chiu, Demler, Merikangas & Walters, 2005). Depression is a chronic and recurrent psychiatric condition that has been characterized by an array of symptoms, which vary between patients (American Psychiatric Association, 2013). The most prominent symptoms include perpetual depressed mood, recurrent thoughts of death and suicide, feeling of worthlessness, social isolation and anhedonia, which lead to a significant decrease in overall quality of life (Jeon, Buettner & Snyder, 2014; McIntyre, Weiller, Zhang & Weiss, 2016; Pu, Luo, Wang, Ju & Lu, 2018). MDD has been determined as the main risk factor in death by suicide (Angst, Angst & Stassen, 1999) and is now considered the second leading cause of disability worldwide (Mathers & Loncar, 2006). Depression presents not only a huge healthcare challenge but also has big social and economic consequences. In the United States alone, the annual cost of MDD is estimated to be around 70 billion dollars (Kessler, 2012), while in Europe, it was estimated to reach 118 billion euros in 2004 (Sobocki, Jönsson, Angst & Rehnberg, 2006). Moreover, according to recent findings of the World Health Organization, MDD affects 12% of the population in the European region and 16% in American Region (World Health Organisation, 2017). Nevertheless, MDD remains elusive and its pathophysiology and aetiology poorly understood.

The majority of current knowledge is based on studies examining the maladaptive changes induced by MDD; meanwhile, the development of therapeutics is aimed at reversing those effects. However, recent research has shifted focus, aiming to understand why certain individuals do not develop MDD despite exposure to stress or traumatic events, a phenomenon referred to as resilience (Schetter & Dolbier, 2011). In this paradigm, importance is put on understanding depression‐induced changes that are adaptive (pro‐resilient). The need for a new approach is evident in the current state of available treatments for MDD, with the majority of them based on serendipitous discoveries made more than 60 years ago. Although many treatments exist, only 30% of patients completely remit and do not experience another depressive episode following treatment with current first‐line antidepressant therapies (Krishnan & Nestler, 2008). Furthermore, it is estimated that about 30–50% of patients are unresponsive to any currently approved treatment (Krishnan & Nestler, 2008). This lack of efficacy suggests that current therapies, mainly targeting neuron‐centric mechanisms, fail to address important biological processes involved in MDD pathology.

MDD has a multifactorial aetiology, with no single established mechanism that can explain all aspects of the disease. This is well reflected in high comorbidity with other chronic physical diseases, including, but not limited to, hypertension, coronary artery disease, diabetes, cerebrovascular disease and chronic pain syndromes (Egede, 2007; Moussavi et al., 2007). Clinical studies have reported that the prevalence of MDD is two‐ to threefold higher in patients suffering from cardiovascular diseases (Ormel et al., 2008; Yapislar, Aydogan & Ozüm, 2012). Concurrently, MDD is linked to a ± 80% increased risk of cardiovascular morbidity (Barth, Schumacher & Herrmann‐Lingen, 2004; Carney, Freedland, Miller & Jaffe, 2002; Ford, Mead, Chang, Cooper‐Patrick & Wang, 1998; Rugulies, 2002; Van der Kooy et al., 2007; van Marwijk, Kooy, Stehouwer, Beekman & Hout, 2015). Similarly, patients with MDD also display higher levels of circulating pro‐inflammatory signals such as cytokines and circulating leukocytes (Dowlati, Herrmann, Swardfager, Liu & Sham, 2010; Lanquillon, Krieg, Bening‐Abu‐Shach & Vedder, 2000; Maes, Meltzer, Bosmans, Bergmans & Vandoolaeghe, 1995; Maes, Stevens, et al., 1992; Maes, Van der Planken, et al., 1992). Interestingly, increased peripheral immune response is particularly exacerbated in treatment‐resistant patients (Kiraly, Horn, Van Dam, Costi & Schwartz, 2017). The majority of those conditions are associated with complex immune responses, from both innate and adaptive systems that can either contribute to their development and progression or, conversely, defend against them.

Epidemiological studies have consistently reported a higher prevalence of MDD in women compared to men (Angst, Gamma, Gastpar, Lepine & Mendlewicz, 2002; Bebbington, Dunn, Jenkins, Lewis & Brugha, 1998; Kuehner, 2003; Silverstein, 1999). Increased incidence for depression in women has been linked to the onset of puberty (Breslau, Gilman, Stein, Ruder & Gmelin, 2017) and persists throughout reproductive years, when oestradiol levels are fluctuating (Deecher, Andree, Sloan & Schechter, 2008). Men and women show differences in important clinical features of depression, such as clinical symptoms, suicide rate and morbidity (Yang, Peng, Ma, Meng & Li, 2017). Depressed women are three to five times more likely to attempt suicide, whereas men with MDD are at higher risk for successful attempts (Oquendo, Ellis, Greenwald, Malone & Weissman, 2001). Symptomatically, MDD women often suffer from comorbid anxiety and report more weight gain and fatigue (Young, Scheftner, Fawcett & Klerman, 1990), while depressed males are more prone to addiction and self‐dislike (Zetin, Sklansky & Cramer, 1984). Gender disparities are also found in tolerability and responsivity to antidepressant treatment (Khan, Brodhead, Schwartz, Kolts & Brown, 2005; Kornstein, Schatzberg, Thase, Yonkers & McCullough, 2000). Women react better to selective serotonin reuptake inhibitors antidepressants (Khan et al., 2005), while men show better response to tricyclic antidepressant treatments (Kornstein et al., 2000). Historically, females have been under‐represented in preclinical and clinical studies. Until the last quarter of the twentieth century, sex was generally not considered as a factor that could affect health and illness. Therefore, only males were used in preclinical and clinical studies, for simplicity and homogeneity purposes, possibly contributing to the high rate of resistance to the current available antidepressant treatments (Uhl, Parekh & Kweder, 2007). In recent years, efforts to reduce the gender imbalance using females and women in MDD research have yielded some significant mechanistic insights into the underlying sex‐specific mechanisms of the disorder.

We propose here a potential causal link between endocrine signals, exacerbated immune response and vascular dysfunction contributing to depression pathogenesis and complexity of sex‐specific symptomatology and treatment. On the other hand, stress resilience may be associated with appropriate coping strategies but also biological adaptations preventing the establishment of depression‐like behaviours in mice and MDD in humans.

## 2 ENDOCRINE PROCESSES INVOLVED IN STRESS VULNERABILITY AND RESILIENCE

### 2.1 Overview of the endocrine system

**Figure 1:**
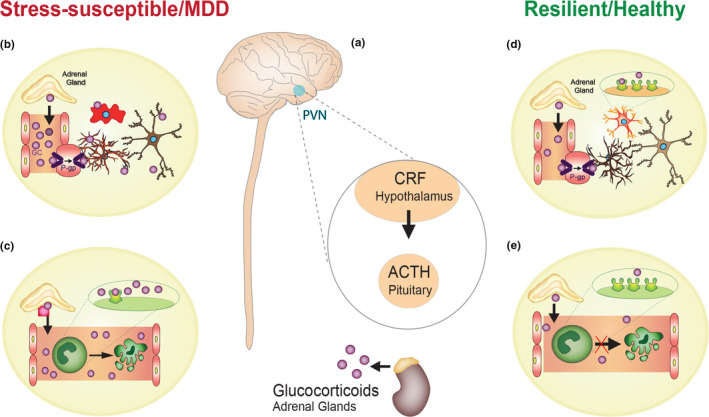
HPA axis‐driven mechanisms promoting stress susceptibility vs resilience. The HPA axis is one of the primary endocrine systems rapidly recruited in order to promote and establish the maintenance of body homeostasis after a challenge or stressor. When exposed to physical and/or psychological stress, neurons from the paraventricular nucleus of the hypothalamus release the CRH. CRH enters blood circulation and reaches the anterior pituitary where it can interact with specific receptors leading to the release of ACTH, which in turn acts in the cortex of the adrenal glands to promote GC release (a). The peripherally released GCs can enter the CNS through P‐gp transporters (purple) present in the endothelial cells that line the BBB. Once into the CNS, GCs can interact with steroid receptors highly expressed in brain cells such as astrocytes (orange), microglia (red) and neurons (brown). The excess of GCs or prolonged binding to these cells can generate an inflammatory status that might contribute to the development of depression (b). In the body periphery, GCs are primarily involved in energy mobilization and use. They also present an anti‐inflammatory effect and can induce macrophage cell apoptosis (green) in order to counteract the inflammatory processes, mechanisms that might be involved in stress susceptibility and MDD (c). On the other hand, during health and resilience, increased number of GC receptors (green) in brain cells (d) and decreased levels of circulating GCs (e) indicate a proper HPA function and cessation. Abbreviations: HPA: hypothalamus–pituitary–adrenal; CRH: corticotropin‐releasing hormone; ACTH: adrenocorticotropic hormone; GC: glucocorticoid; CNS: central nervous system; P‐gp: P‐glycoprotein transporters; BBB: blood–brain barrier; MDD: major depressive disorder

Appropriate response to acute stress, a crucial process for survival in life‐threatening situations, is mediated through parallel activity of the autonomic nervous system (ANS) and the hypothalamic–pituitary–adrenal (HPA) axis (Herman, Mcklveen, Ghosal, Kopp & Wulsin, 2016; Smith & Vale, 2006) (see Figure 1). These processes are regulated by neural circuitry in the hypothalamus, brainstem and forebrain (Ulrich‐Lai & Herman, 2009). Upon receiving a danger signal, the activation of the ANS promotes a rapid physiological response that involves the coordinated activity of sympathetic and parasympathetic signalling. Initial activation of spinal preganglionic sympathetic neurons leads to subsequent activity in pre‐ or paravertebral ganglionic neurons that regulate function of cardiovascular and visceral organs as well as the adrenal medulla. Stress‐induced ANS activation induces release of epinephrine from the adrenal medulla and noradrenaline from sympathetic nerves, resulting in modulation of heart rate, blood pressure and vasoconstriction (Burford, Webster & Cruz‐Topete, 2017). Concurrent activation in the HPA axis leads to secretion of corticotropin‐releasing factor (CRF) from the paraventricular nucleus of the hypothalamus. This in turn stimulates the release of anterior pituitary gland hormone adrenocorticotropin (ACTH) and subsequent production and release of glucocorticoids (GCs) from the adrenal cortex (Herman et al., 2016) (see Figure 1). GCs primary function in the periphery is to promote mobilization and utilization of energy and mineral reserves during stress (Garabedian, Harris & Jeanneteau, 2017). In the brain, GCs interact with steroid receptors, such as glucocorticoid and mineralocorticoid receptors that are highly expressed in the hippocampus, amygdala, prefrontal cortex and other limbic and midbrain structures (Mahfouz, Lelieveldt, Grefhorst, Weert & Mol, 2016; Morimoto, Morita, Ozawa, Yokoyama & Kawata, 1996; Wang, Verweij, et al., 2014; Wang, Pinol, Byrne, and Mendelowitz, 2014). Their activation is responsible for modulation of neural circuitry and neuroendocrine systems underlying behavioural responses to stress (see Figure 1). These receptors function primarily as transcription factors and are responsible for modification of cellular mechanisms, such as altering gene expression and affecting synaptic plasticity, beyond the time scale of acute stress effects. There is abundant crosstalk between the ANS and the HPA, designed to properly tune the adaptive response to stress and maintain physiological homeostasis (Herman et al., 2016). Understanding the peripheral and central effects of HPA and ANS activation in different preclinical models of depression and groups of MDD patients based on their symptomatology could help clarify the mechanisms contributing to this disorder and improve therapeutical strategies.

### 2.2 Role of the endocrine system in depression and stress resilience

Failure to resolve a physiological response upon cessation of an acute stressful stimulus can lead to the creation of deleterious allostatic load defined as a cumulative burden of adaptations placed upon the brain and body (Goldstein, Mcewen & Section, 2002). This in turn results in stress vulnerability and increased risk of neuropsychiatric disorders, including MDD (Charney, 2004; Goldstein et al., 2002; McEwen, Bowles, Gray, Hunter & Karatsoreos, 2015). Indeed, chronic stress is among the most important risk factor for MDD, with HPA axis dysfunction, GCs resistance as well as disrupted HPA–ANS crosstalk being a hallmark of MDD pathology (Hammen, 2005; Straub, Buttgereit & Cutolo, 2011). A growing body of evidence shows elevated blood levels of GCs in about two‐thirds of patients with MDD (Juruena, Cleare, Papadopoulos, Poon & Lightman, 2006; Pariante, 2009) (see Figure 1). Interestingly, a meta‐analysis study reported that a small subset of MDD individuals is characterized by low GC levels and less severe depressive symptoms (Stetler & Miller, 2011) (see Figure 1). The same work also revealed that, despite the fact that these MDD patients presented elevated cortisol and ACTH levels, CRF secretion is unchanged. It seems that age is positively correlated with HPA outcome (Stetler & Miller, 2011). This is in line with studies reporting cortisol level differences between depressed and non‐depressed subjects in older individuals when compared with studies focusing on younger patient cohorts (Stetler & Miller, 2011). In addition, sex seems to have an influence on the HPA axis, and these differences can be observed both in baseline and during stress and depression as discussed in the next section (Babb, Masini, Day & Campeau, 2013; Handa & Weiser, 2014; Iwasaki‐Sekino, Mano‐Otagiri, Ohata, Yamauchi & Shibasaki, 2009; Kitay, 1961; Lundberg, Martinsson, Nylander & Roman, 2017; Viau, Bingham, Davis, Lee & Wong, 2005; Weinstock, Razin, Schorer‐Apelbaum, Men & McCarty, 1998).

Nonetheless, in the majority of individuals, stressful events evoke adaptive coping mechanisms, occurring in both the brain and periphery that promote resilience (Pfau & Russo, 2015; Russo, Murrough, Han, Charney & Nestler, 2012). In agreement with this observation, preclinical studies using animal models of depression (see Box 1) indicate that modulation of the HPA axis function during stress response can promote a resilient phenotype (Plotsky & Meaney, 1993; Weaver et al., 2005). For instance, findings from rodent studies show a decrease in CRF release in response to stress in adult individuals subjected to postnatal handling, a model defined as mild‐to‐moderate early‐life stress (Plotsky & Meaney, 1993) (Table 1). In addition, rats exposed to a more severe stressor, such as maternal separation, displayed higher CRF hormone expression when compared to postnatal non‐handled rats or those exposed to a mild‐to‐moderate early‐life stress (Plotsky & Meaney, 1993). Such discrepancies have been associated with diverse levels of maternal care displayed by mothers of handled and non‐handled rodents. Indeed, abundant maternal behaviours, including frequent licking, grooming and arched back nursing of pups, have been found to negatively affect repressive DNA methylation of the GC receptor gene promoter. This in turn leads to increased expression of GC receptors in the hippocampus and, subsequently, enhanced sensitivity to GC‐negative feedback resulting in better stress adaptation (Weaver et al., 2005) (see Figure 1). A similar phenomenon has been reported by clinical studies in patients suffering from post‐traumatic stress disorder, where peripheral high‐dose administration of GCs had pro‐resilient effects (Kearns, Ressler, Zatzick & Rothbaum, 2012; Schelling, Roozendaal, Krauseneck, Schmoelz & Quervain, 2006; Surís, North, Adinoff, Powell & Greene, 2010).

**Table 1:** Behavioural outcomes for animal models of depression

Animal model	Behaviour
Chronic mild stress or chronic unpredictable stress	↑Anhedonia, ↑Sleep disturbance, ↑Immobility (forced swim test, tail suspension test), ↓Grooming, ↓Weight
Learned helplessness	↓Active avoidance, ↓Weight, ↑Sleep disturbance
Chronic social defeat stress (CSDS)*	↑Anhedonia, ↑Sleep disturbance, ↓Exploratory anxiety, ↓Weight
Repeated social defeat (RSD)*	↑ Anxiety, ↑ Social avoidance, ↓Weight
Early‐life stress	↑ Anhedonia, ↑ Anxiety, ↑ Depressive‐like behaviour in adulthood, ↓ Learning, ↓ Locomotor activity (Open field test)
Lipopolysaccharide‐induced sickness behaviour	↑Anhedonia, ↑Lethargy, ↓Appetite and food intake, ↑Anxiety

* CSDS and RSD models were labelled as “social stress” and “psychosocial stress”, respectively.

Box 1 **Animal models of mood disorders**

1 Animal models of depression offer key insights into understanding the unique and complex pathogenesis of mood disorders. However, producing animal models of mental illness proves to be difficult, due to the lack of objective tests and reliable biomarkers, as well as the personal and verbal nature of the symptoms (e.g. sadness, guilt, feeling of worthlessness) which cannot be assessed in animals (for review, see Nestler & Hyman, 2010). A growing number of animal models showing great predictive validity have been developed and widely used to induce behavioural changes qualified as ‘depressive‐like’ symptoms (see Table 1), in line with those observed in depressed humans.

**Chronic mild stress (CMS)**

This model, also called chronic unpredictable stress (CUS), focuses on a core symptom of depression, anhedonia. Animals are subjected to a variety of mild stressors, such as tail suspension, tube restraint or periods or food and water deprivation, and stressors are alternated to prevent habituation. Most protocols can last for weeks, although six days of CMS is enough to see sex differences (Hodes et al., 2014).

Animals subjected to CMS show peripheral pro‐inflammatory immune activation, namely an increase in cytokines IL‐1β, IL‐6 and TNF‐α. Symptoms can be reversed by chronic, but not acute antidepressant exposure (Willner, 1997). To our knowledge, CMS‐induced vascular changes have yet to be determined.

**Learned helplessness (LH)**

Proposed by Seligman and colleagues, LH is a widely used model to study coping mechanisms and depressive disorders (Maier & Seligman, 1976; Overmier & Seligman, 1967; Seligman & Beagley, 1975). In this paradigm, animals are exposed to uncontrollable stressful events, such as tail or foot shocks. Thereafter, they are reintroduced into the same environment, but with the possibility to escape the stressful event. Most of previously stressed animals will not learn how to escape this negative event, which is characterized as ‘helplessness’ and indicates a ‘depressive‐like’ phenotype. Animals show an increase in pro‐inflammatory cytokine levels, such as IL‐1β, IL‐6, TNF‐α, INF‐γ and G‐CSF, and vascular adaptations (Cheng et al., 2015, 2018). Antidepressant treatment reverses those behaviours in 3 to 5 days.

The validity of the LH model to study sex differences has been questioned since studies report that, unlike male rats, females learn to escape the stressful task, thus not expressing helplessness behaviour (Heinsbroek, Haaren, Poll & Steenbergen, 1991; Kirk & Blampied, 1985; Steenbergen, Heinsbroek, Hest & Poll, 1990). Other studies have found LH behaviour to be independent of gonadal hormones exposure during adulthood, as neither ovariectomy nor castration, abolished those differences in LH behaviour, making them question the validity of this paradigm to study female rats (Dalla, Edgecomb, Whetstone & Shors, 2008; Pryce, Azzinnari, Spinelli, Seifritz & Tegethoff, 2011).

Chronic social defeat stress (CSDS)*

In this paradigm, over the course of 10 days, animals are subjected to bouts of social defeat by a larger CD‐1 mouse screened for aggressive behaviour. About two‐thirds of animals, termed stress‐susceptible (SS), develop marked social avoidance, assessed by the social interaction (SI) test. The remaining one‐third of animals, termed ‘resilient’, fails to develop social avoidance or anhedonia and behave similarly to controls (Golden et al., 2011).

Depressive‐like phenotype is characterized by increased pro‐inflammatory response and region‐specific BBB hyperpermeability, while resilient mice show less pro‐inflammatory activation and intact BBB integrity. Social avoidance can be reversed by a chronic, but not acute antidepressant treatment, although IL‐6 levels remain elevated in susceptible mice after such treatment (Berton, McClung, Dileone, Krishnan & Renthal, 2006).

A major limitation of this paradigm was its inability to be implemented in female mice. Indeed, under most conditions, the resident mice will not attack a female intruder mouse, limiting the inclusion of female subjects in this paradigm. Harris and colleagues have recently developed an accessible and valuable model for chronic social defeat in female mice. Applying CD1 male urine to the vaginal orifice as well as at the base of the tail is enough to replicate bullying behaviour similar to those observed in male subjected to CSDS (Harris, Atsak, Bretton, Holt & Alam, 2018; Toyoda, 2017). Similarly, Takahashi et al. showed that male aggression towards females through chemogenetic activation of the ventrolateral subdivision of the ventromedial hypothalamus induces social avoidance, anxiety‐like behaviours, reduction of body weight and elevated circulating levels of IL‐6 in female mice (Takahashi, Chung, Zhang, Zhang & Grossman, 2017).

Repeated social defeat (RSD)*

In the RSD paradigm, C57BL/6 mice are subjected to aggressive behaviour for 2 h over six consecutive nights by a novel, dominant CD‐1 mouse, introduced into their home cage. This disrupts the social hierarchy of the cage and eliciting submissive behaviours of C57BL/6 mice (Avitsur & Sheridan, 2009). Male mice show an increase in circulating IL‐6 levels (Wohleb, Patterson, et al., 2014; Wohleb, McKim, et al., 2014).

**Early‐life stress (ELS)**

Animals are subjected to early‐life adversity, the most common manipulation being maternal separation (MS). The stressor lasts for few hours a day, during the first postnatal weeks of life. Then, in adulthood, animals are tested for increased anxiety or susceptibility to depressive‐like behaviours.

Many reports attest that this model increases resilience in adulthood (Daniels, Pietersen, Carstens & Stein, 2004; Huot, K., Meaney & Plotsky, 2001; Kalinichev, Easterling, Plotsky & Holtzman, 2002; Lee, Kim, Kim, Ryu & Kim, 2007; Peña, Kronman, Walker, Cates & Bagot, 2017; Romeo, Mueller, Sisti, Ogawa & McEwen, 2003), while others show no behavioural effects (Lehmann, Pryce, Bettschen & Feldon, 1999; Millstein & Holmes, 2007; Savignac, Dinan & Cryan, 2011). These discrepancies might be due to variations in the experimental conditions between studies (for review, see Murthy & Gould, 2018).

**Lipopolysaccharide (LPS)‐induced sickness behaviour**

LPS is a cell membrane component of gram‐negative bacteria and is a potent inducer of inflammatory response. Its injection is used to challenge the rodent's immune machinery (Bassi, Kanashiro, Santin, Souza & Nobre, 2012). LPS has been widely studied for its ability to generate profound physiological and behavioural changes, also known as ‘sickness behaviour’ (Dantzer et al., 2008; Dantzer, 2001; Konsman, Parnet & Dantzer, 2002). After LPS challenge, excessive secretion of pro‐inflammatory mediators, including TNF‐α (Rothe, Lesslauer, Lötscher, Lang & Koebel, 1993; Tracey, Beutler, Lowry, Merryweather & Wolpe, 1986) and IL‐6 (Chai, Gatti, Toniatti, Poli & Bartfai, 1996), by macrophages has been observed (Freudenberg, Keppler & Galanos, 1986).

Limitations of the LPS model include the rapid resolution of the symptoms, usually within 24 h, and its systemic administration occasionally results in tolerance (Barr, Song, Sawada, Young & Honer, 2003). LPS injection also induces global hyperalgesia (Suzuki & Nakano, 1986), which decreases the sensibility of this paradigm, as more than one variable is modulated (for review, see Pitychoutis, Griva, et al., 2009; Pitychoutis, Nakamura, et al., 2009).

*To simplify reading, CSDS and RSD models were labelled as ‘social stress’ and ‘psychosocial stress’, respectively.

Another endocrine system involved in stress and MDD pathogenesis is the locus coeruleus (LC)‐norepinephrine (NE) system. The LC is located in the pons and is composed mostly of NE neurons that send widespread efferent projections to the entire neuroaxis (Aston‐Jones, 2004). Most of the NE in the central nervous system (CNS) is synthetized and released from neurons of the LC. The LC‐NE system is responsible for modulating arousal, cognition and attention during behaviour (Schwarz & Luo, 2015). This neurotransmitter is also a known regulator of immune function by acting on α‐ and β‐adrenergic receptors in the plasma as well as on β‐adrenergic receptors in the brain (Johnson, Campisi, Sharkey, Kennedy & Nickerson, 2005). In the periphery, β‐adrenergic receptors are found on the cell surface of macrophages and neutrophils of both rodents and humans. Activation of these receptors by NE stimulates the release of pro‐inflammatory cytokines in rodents (Finnell, Moffitt, Hesser, Harrington & Melson, 2019; Flierl, Rittirsch, Nadeau, Sarma & Day, 2009; Li, Yao, Li & Xi, 2015). However, in some pathological conditions, the activation of β‐adrenergic receptors can decrease pro‐inflammatory cytokine levels, especially tumour necrosis factor‐alpha (TNF‐α) in the periphery (Bosmann, Grailer, Zhu, Matthay & Sarma, 2012; Nijhuis, Olivier, Dhawan, Hilbers & Boon, 2014; Walker, Anderson, Jiang, Bahouth & Steinle, 2011). NE also plays a crucial role in central neuroinflammatory processes, including modulating microglial motility and function (Gyoneva & Traynelis, 2013; Jardanhazi‐Kurutz, Kummer, Terwel, Vogel & Thiele, 2011; Johnson et al., 2005), further suggesting interaction between endocrine mechanisms, peripheral and central inflammation. Recent evidence suggests that the central NE system favours bidirectional communication with the cardiovascular system, as well as playing an important role in the cardiovascular consequences of stress. For example, during hypotensive stress in rats, endogenous CRF promotes activation of LC neurons (Valentino, Page & Curtis, 1991). Conversely, optogenetic stimulation of LC neurons provokes an increase in the frequency of inhibitory postsynaptic currents in cardiac vagal neurons (Wang, Verweij, et al., 2014; Wang, Pinol, et al., 2014). Sustained activation of the brain NE system, in parallel with HPA axis hyperactivity, is now considered a hallmark of chronic stress response (Wood, Valentino & Wood, 2017). Although beyond the scope of this review, better understanding the links between the LC‐NE, the immune and the cardiovascular system could give mechanistically relevant insights into susceptibility and resilience to stress. Thus, a shift in scientific effort to better understand the mechanisms underlying appropriated behavioural and physiological stress response, including relationships between stress‐evoked endocrine, immune and vascular adaptations, would be highly valuable to the field and potentially provide novel therapeutic strategies for treatment‐resistant depressed patients.

### 2.3 Sex differences in the endocrine system stress responses

Sex differences in the HPA axis have been extensively documented and have been suggested to drive sexually dimorphic stress responses. At baseline, female rodents display a more active HPA axis than their male counterparts, as well as higher circulating levels of corticosterone (Kitay, 1961; Viau et al., 2005; Weinstock et al., 1998). Following acute stress, female rodents display a more robust and prolonged activation of the HPA axis (Babb et al., 2013; Handa & Weiser, 2014; Iwasaki‐Sekino et al., 2009) and a delayed return to baseline ACTH and corticosterone levels compared to males, highlighting sex differences in the negative feedback regulation of the HPA axis (Babb et al., 2013; Iwasaki‐Sekino et al., 2009; Viau et al., 2005). Studies conducted in rats suggest heightened CRF sensitivity in females as compared to males (Bangasser et al., 2010, 2013). Release of limbic CRF can modulate HPA axis activity and monoamine systems implicated in mood and cognition (Rodaros, Caruana, Amir & Stewart, 2007; Valentino & Van Bockstaele, 2008; Wanat, Hopf, Stuber, Phillips & Bonci, 2008). Increased CRF sensitivity in female rats was associated with sex differences in CRF1 receptor signalling and trafficking in the LC‐NE system, decreasing their ability to adapt to chronic stressors (Bangasser & Valentino, 2012; Bangasser, Curtis, Reyes, Bethea & Parastatidis, 2010; Curtis, Bethea & Valentino, 2006) (see Table 2). To better understand the importance of sex differences in anxiety and depression, Kokras and colleagues performed adrenalectomy with corticosterone replacement in male and female rats. When subjected to forced swim and open field tests, adrenalectomized males show reduced anxiety‐like behaviour compared to sham‐operated controls, a difference that was not observed in adrenalectomized females (Kokras, Dalla, Sideris, Dendi & Mikail, 2012). Thus, male, but not female behavioural responses seem to be affected by adrenalectomy in stress paradigms, suggesting involvement of sex‐specific mechanisms in stress regulation. These findings play a significant role in understanding differential coping strategies between sexes; nevertheless, further studies are necessary to elucidate the mechanisms involved.

**Table 2:** Summary of sex‐specific alterations in stress susceptibility and resilience in rodents

Field	Adaptation (♀ vs ♂)	References
Neuroendocrine	More active HPA axis and ↑ corticosterone levels at baseline	Weinstock et al. (1998)
	↑ CRF sensitivity at baseline	Curtis et al. (2006); Bangasser et al. (2010)
	↑ ACTH sensitivity in adrenal cortex at baseline	Viau and Meaney (1991);
	↑ ACTH levels and CRF mRNA levels in PVN at baseline	Iwasaki‐Sekino et al. (2009)
	Prolonged HPA activation following acute stress	Viau et al. (2005); Iwasaki‐Sekino et al. (2009); Babb et al. (2013)
Immune (periphery)	↑ Phagocytic activity of neutrophils and macrophages	Spitzer (1999)
	Following LPS administration:	Aomatsu et al. (2013)
	↑ phagocytic capacity of neutrophils (following LPS administration)	
	↓ TLR4 on macrophages and neutrophils	
	Following chronic stress (in both ♂ and ♀):	Wohleb, Patterson, et al. (2014), Wohleb, McKim, et al. (2014), McKim, Weber, et al. (2018), McKim, Yin, et al. (2018), Yin et al. (2019); Pitychoutis, Griva, et al. (2009), Pitychoutis, Nakamura, et al. (2009)
	↑Myelopoiesis, monocyte and granulocyte accumulation in blood and spleen Microglial hyperreactivity Monocyte recruitment to the brain ↓ NK cell activity (in ♀)	
Immune (central)	↑Neuroprotective phenotype of microglia (transcriptome)	Villa et al. (2018)
	↑ Proportions of primed to ramified microglia in the PFC at resting state	
	↑ CX3CL1 to CX3CR1 signalling at resting state	
	↓ Proportions of primed microglia following chronic restraint stress (see Box 1)	Bollinger et al. (2016)
	↑ Baseline levels of TNF‐α, IL‐1β, IL‐6 and IL‐10 mRNA levels (postnatal day 3)	Crain et al. (2013)
	↓ Numbers and processes complexity of astrocytes in posterodorsal portion of the medial amygdala	Johnson et al. (2008)
Vascular^a^	↑ Oestradiol = ↑ transendothelial resistance *in vitro* in naïve animals	Burek et al. (2010)

^a^ No data on sex‐specific adaptations in depression and/or resilience.

Conversely, in healthy humans, cortisol levels are typically comparable between men and women at baseline conditions (Kirschbaum, Kudielka, Gaab, Schommer & Hellhammer, 1999; Uhart, Chong, Oswald, Lin & Wand, 2006). However, women with depressive symptoms tend to have higher cortisol levels than depressed men (Young & Altemus, 2004; Young, 1995), which has been attributed to impaired GC‐negative feedback of the HPA axis (Reul & de Kloet, 1985). Elevated levels of CRF were also found in the cerebrospinal fluid (de Bellis, Gold, Geracioti, Listwak & Kling, 1993; Heuser, Bissette, Dettling, Schweiger & Gotthardt, 1998; Nemeroff, Widerlöv, Bissette, Walléus & Karlsson, 1984) and in postmortem brain samples of MDD patients (Austin, Janosky & Murphy, 2003; Bissette, Klimek, Pan, Stockmeier & Ordway, 2003; Raadsheer, Hoogendijk, Stam, Tilders & Swaab, 1994; Wang, Kamphuis, Huitinga, Zhou & Swaab, 2008). Nevertheless, few studies have considered sex differences in regard to CRF sensitivity in humans.

There is a large body of evidence linking higher depression susceptibility and HPA axis hyperactivity in females to the organizational and activational effects of gonadal hormones in rodent models of depression (Atkinson & Waddell, 1997; Seale, Wood, Atkinson, Harbuz & Lightman, 2005; Seale, Wood, Atkinson, Lightman & Harbuz, 2005; Viau & Meaney, 1991). These studies are highly relevant, considering the complex interplay between neural tissues and the endocrine systems. Gonadal hormone receptors are widely expressed in the HPA axis circuitry, enabling gonadal steroids to alter neuroendocrine responses to stress (Handa & Weiser, 2014). For example, androgen and oestrogen receptors are expressed in the cortex, hippocampus, paraventricular nucleus (PVN), hypothalamic areas and adrenal glands (Bentvelsen, McPhaul, Wilson, Wilson & George, 1996; Cutler, Barnes, Sauer & Loriaux, 1978; Green, Dahlqvist, Isenberg, Strausbaugh & Miao, 1999; Sar, Lubahn, French & Wilson, 1990; Shughrue, Lane & Merchenthaler, 1997; Simerly, Swanson, Chang & Muramatsu, 1990), suggesting that gonadal receptors can alter neuroendocrine responses to stress. Sex differences in HPA activity were found to be highly influenced by oestradiol fluctuations during oestrous cycle in rodents. Indeed, oestradiol potentiates stress‐induced neuronal activation in the PVN (Iwasaki‐Sekino et al., 2009; Rhodes, Kennell, Belz, Czambel & Rubin, 2004; Viau & Meaney, 1991) and increases ACTH sensitivity in the adrenal cortex of female rodents. This could explain higher corticosterone secretion in female's neuroendocrine system compared to males following stress (Figueiredo, Ulrich‐Lai, Choi & Herman, 2007). In humans, findings related to stress response of women in varying phases of the menstrual cycle reinforces a role for oestrogen and progesterone in regulating behaviours (Herrera, Nielsen & Mather, 2016). However, discrepancies exist and can emerge from many factors such as age, overall health and menstrual phase of the subjects. Furthermore, male hormones including testosterone are powerful neuromodulators and testosterone level was associated with dominance vs subordination and, thus, coping strategies. Sex differences in stress response and depression should not only be viewed in a frame of hormonal and gonadal differences. Recent data have shown extensive sex differences in transcriptional profiles of chronically stressed mice as well as in MDD patients when compared to healthy controls (Hodes, Pfau, Leboeuf, Golden & Christoffel, 2014; Labonté, Engmann, Purushothaman, Menard & Wang, 2018). These changes are seen across various networks, such as RNA post‐transcriptional modifications or molecular transport. Such a small overlap between differentially expressed genes between the sexes, both in animal models and in MDD patients, shows how important it is to study MDD as part of a systemic homeostasis taking sex as a biological variable.

In this section, we summarized key components and processes occurring in the neuroendocrine system in stress susceptibility and resilience. Throughout the years, a large number of studies have unveiled important neuroendocrine mechanisms driving stress response, involving the ANS and HPA axis. Findings from preclinical studies were often successfully reproduced in human subjects, such as findings of dysregulated HPA axis (Juruena et al., 2006; Pariante, 2009; Stetler & Miller, 2011) or pro‐resilient effects of high GC doses (Kearns et al., 2012; Schelling et al., 2006; Surís et al., 2010). Failure to develop novel drugs targeting HPA axis and neuroendocrine pathways to successfully treat MDD stems from many factors. First of all, to this day, most studies investigating HPA axis response to chronic stress were conducted in male rodents (Beery & Zucker, 2011). As described by Kokras *et al* in a recent review, these data can hardly be translated to human clinical studies, as conclusions cannot be generalized to such a sexually differentiated disorder (Kokras & Dalla, 2017; Kokras, Hodes, Bangasser & Dalla, 2019). However, with several funding agencies now actively encouraging to consider sex as a biological variable in preclinical and clinical research, this imbalance will hopefully be reversed in the years to come (McCullough, Vries, Miller, Becker & Sandberg, 2014). Furthermore, based on the data discussed herein, we suggest that studies considering exclusively the endocrine system in the context of stress susceptibility vs resilience possibly ignore mechanistically important aspects of MDD. As described extensively in this review, growing evidence suggests that MDD is not only neuron‐centric, but a whole body disease involving immune and vascular adaptations at least in subpopulations of depressed individuals. While the endocrine system could play a central role in driving these changes, it is imperative to identify how it affects immune and vascular responses to chronic stress. Emerging data on the LC‐NE system seem to be promising in linking these together. This neuroendocrine system is known to be involved in regulating immune function, in rodent models, by acting on peripheral and central adrenergic receptors (Johnson et al., 2005), modulating microglial motility and function (Gyoneva & Traynelis, 2013; Jardanhazi‐Kurutz et al., 2011; Johnson et al., 2005), as well as priming plasma and brain cytokine release in response to social stress (Finnell et al., 2019). The NE system favours bidirectional communication with the cardiovascular system and plays an important role in the cardiovascular consequences of stress (Valentino et al., 1991; Wang, Verweij, et al., 2014; Wang, Pinol, et al., 2014). This system was also found to be sexually differentiated (Bangasser & Valentino, 2012; Bangasser et al., 2010; Curtis et al., 2006). Although this is only one example, we strongly think there is ample crosstalk between endocrine, immune and neurovascular systems and that further studies should focus on deciphering their complex interactions with each other, in both sexes. This could hold the key to developing more effective treatments for MDD patients worldwide.

## 3 IMMUNOLOGICAL RESPONSE ASSOCIATED WITH DEPRESSION AND RESILIENCE TO STRESS

### 3.1 Overview of the peripheral immune system

There has been an emerging interest in recent years in unravelling the link between chronic stress, maladaptive immune responses and MDD. Inflammation is a natural and complex response of the body, triggered by various harmful stimuli. Immune cells quickly react to prevent inflammation and modulate wound healing as well as maintaining systemic homeostasis (for review, see Liu, Wang & Jiang, 2017). The immune system is comprised of two major components: the innate and the adaptive immune responses. In the following sections, we will provide an overview of these systems and discuss recent findings related to stress vulnerability and resilience including sex differences.

The innate arm of the immune system increases protection offered by anatomical and physiological barriers, such as intact skin, low stomach pH or saliva. It is a highly conserved system, with similar features found in plants, invertebrates and mammals (Bryant & Monie, 2012; Buchmann, 2014). Innate immunity represents the first line of defence of the organism, by mounting an all‐purpose and rapid response that provides protection against danger signals. This system is mostly, but not exclusively, derived from hematopoietic stem cells originating from bone marrow niches (Scheiermann, Frenette & Hidalgo, 2015). It includes myeloid cells, such as granulocytes, monocytes, macrophages and dendritic cells (DCs) (Hashimoto, Miller & Merad, 2011), as well as innate lymphoid cells, like natural killer (NK) cells (Spits, Artis, Colonna, Diefenbach & Santo, 2013). The innate immune response relies on readily available cells equipped with pattern recognition receptors (PRRs), including, among others, toll‐like receptors. PRRs recognize pattern recognition pathogen‐associated molecular patterns or cell‐derived damage‐associated molecular patterns (Frank, Watkins & Maier, 2013; Portou, Baker, Abraham & Tsui, 2015). Activation of the innate response results in fast release of inflammatory mediators, such as prostaglandins, bradykinin, histamines and serotonin, leading to recruitment of immune cells to the site of injury and release of pro‐inflammatory cytokines by macrophages and tissue‐resident DCs (Portou et al., 2015; Rosenblat, Cha, Mansur & McIntyre, 2014).

**Figure 2:**
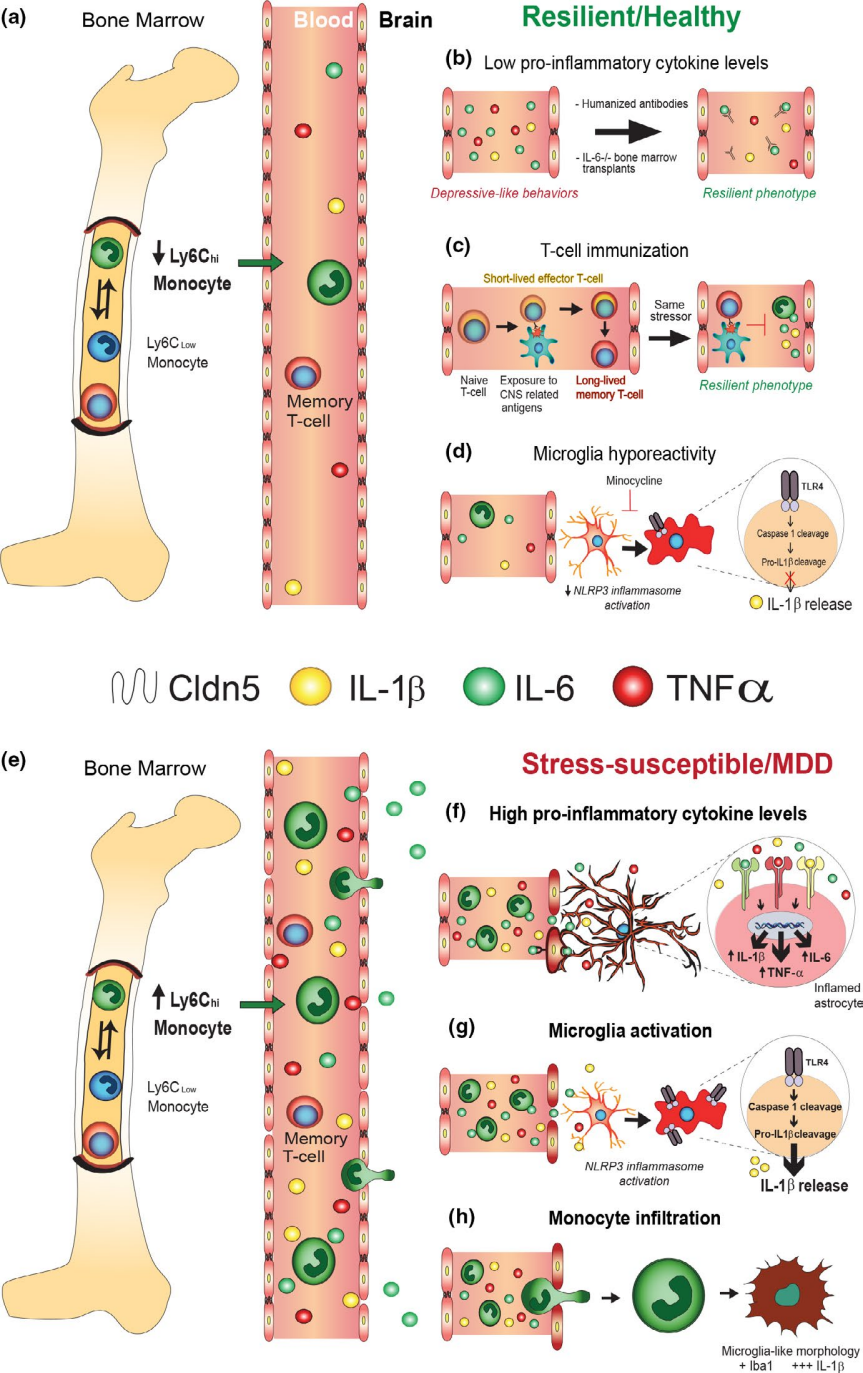
Immune mechanisms affecting blood–brain barrier permeability in stress vulnerability and resilience. Chronic stress may be sufficient to mobilize deleterious activation of the innate immune system driving increased proliferation and release of inflammatory Ly6C^high^ monocytes (a, e) and neutrophils. Resilient phenotype is characterized by lack of exacerbated immune responses following acute or chronic stressors. Moreover, depressive‐like behaviour has been shown to be reversed due to the decrease in circulating pro‐inflammatory cytokines levels by the antidepressant treatment, humanized antibodies or IL‐6^−/−^ bone marrow transplants (b). In comparison, susceptible animals and MDD patients display increase in circulating levels of pro‐inflammatory cytokines (IL‐1β and IL‐6) in blood leading to astrocyte activation and a deleterious cascade of pro‐inflammatory cytokine production (f). Animal studies have shown that T‐cell‐dependent immunization to CNS‐related antigens prior to the exposure to chronic mild stress ameliorated subsequent depression‐like behaviours (c). Minocycline, a microglial activation inhibitor, abolishes stress‐induced hyper‐ramification and reverses depression‐like behaviours (d). This would suggest that lowering microglia reactivity as well as NLRP3 inflammasome activation, highly abundant in microglia, could contribute to resilient phenotype. On the other hand, chronic stress induces pro‐ramifying effect on microglia. For instance, TLR4‐induced activation of NLRP3 inflammasome leads to production and secretion of pro‐inflammatory cytokine IL‐1β (g). Microglia‐derived cytokine, CCL2, attracts patrolling immature Ly6C^high^ monocytes, which can cross the BBB and penetrate into the brain parenchyma (h). In stress‐related brain regions, these monocytes could differentiate into phagocytic macrophages positive for microglia marker Iba1. Abbreviations: BBB: blood–brain barrier, CCL2: chemokine ligand 2, CNS: central nervous system, Iba1: ionized calcium binding adaptor molecule 1, IL‐1β: interleukin‐1β, IL‐6: interleukin‐6, Ly6C^high^monocytes: lymphocyte Ag 6C high monocytes, MDD: major depressive disorder, NLRP3 inflammasome: nucleotide‐binding domain and leucine‐rich repeat protein‐3 (NLRP3) inflammasome, TLR4: toll‐like receptor 4

In response to both psychological and physiological stress, neutrophils and immature Ly6C^hi^ monocytes increase in number from hematopoietic stem cells and rapidly reach tissues through the bloodstream (Ginhoux & Jung, 2014) (see Figure 2). Once they are recruited into the tissues, Ly6C^hi^ monocytes can further differentiate into mononuclear phagocytes, such as macrophages and DCs, to enhance inflammatory processes or promote resolution of inflammation (Ginhoux & Jung, 2014; Shi & Pamer, 2011).

In contrast to the innate immune system, the adaptive arm of the immune system can recognize and remember a wide range of antigens, depending upon T and B lymphocytes, by triggering a targeted and enhanced immune response to antigen encounters (Rainville, Tsyglakova & Hodes, 2018). Effector memory T cells circulate in the bloodstream, providing immunosurveillance, while central memory T cells reside mainly in secondary lymphoid organs, such as lymph nodes and spleen in order to establish immunological memory (Nakai, Hayano, Furuta, Noda & Suzuki, 2014). Upon presentation of a previously encountered antigen by an antigen‐presenting cell, memory T cells are activated and proliferate. Tissue‐resident memory T cells rapidly initiate cytokine release as well as recruitment of DCs and NK cells (Mueller & Mackay, 2016). Contribution from both peripheral innate and adaptative immune systems has been demonstrated to be involved in MDD; thus, the discovery of the mechanisms that lead to exacerbated immune responses in the pathophysiology of depression is extremely relevant for the field.

### 3.2 Overview of the central immune system

In the CNS, the immune machinery is mainly composed of microglia, which represent 10–15% of all brain cells. Originating from the yolk sac, they constitute the tissue‐resident macrophages of the brain (Ransohoff & Brown, 2012) and are essential in immune‐related functions as well as brain development (Kreutzberg, 1996; Rosen, Ham & Mogil, 2017). Once established in the CNS parenchyma, microglia are sustained by proliferation of resident progenitors throughout adult life (Ajami, Bennett, Krieger, Tetzlaff & Rossi, 2007). Under normal conditions, microglia are highly dynamic cells that survey the surrounding environment (Nimmerjahn, Kirchhoff & Helmchen, 2005) and promote recruitment of monocytes to injured tissues to help manage local inflammation (Ajami, Bennett, Krieger, McNagny & Rossi, 2011; Yamasaki, Lu, Butovsky, Ohno & Rietsch, 2014). In healthy subjects, monocytes, DCs and lymphocytes from the periphery can enter the brain through blood–brain barrier (BBB)‐deprived areas, such as the choroid plexus and the circumventricular organs, and communicate with central immune cells (Baruch & Schwartz, 2013; Louveau, Smirnov, Keyes, Eccles & Rouhani, 2015; Shechter, London & Schwartz, 2013).

Astrocytes are another type of glial cells that play an important role in protecting the brain. They are derived from neural stem cells and represent around 20–40% of all glial cells in the CNS (Sofroniew & Vinters, 2010). They perform many functions, such as controlling immune responses, neuronal development and maintaining BBB integrity (Eroglu & Barres, 2010; Farina, Aloisi & Meinl, 2007; Lampron, ElAli & Rivest, 2013). Astrocytes are found surrounding blood vessels and synapses, and they respond actively to inflammatory signals. They can produce and secrete various cytokines, including interleukin‐1β (IL‐1β), interleukin‐3 (IL‐3), interleukin‐6 (IL‐6), TNF‐α and interferon‐γ (IFN‐γ) as well as chemokines, such as chemokine ligand 2 (CCL2) and fractalkine (CX3CL1) (Farina et al., 2007; Lampron et al., 2013). Moreover, astrocytes closely interact with endothelial cells, providing crucial support to maintain the restricted permeability of the BBB (Lampron et al., 2013). In pathological conditions or following insult to the CNS, astrocytes respond through astrogliosis and glial scar formation in order to restore brain homeostasis (Sofroniew & Vinters, 2010). Astrogliosis constitutes a spectrum of dynamic modifications, such as upregulation of glial fibrillary acidic protein (GFAP), a mature astrocyte marker, cellular hypertrophy and altered gene expression. Pro‐inflammatory cytokines, such as IL‐1β, TNF‐α, IFN‐γ and TGF‐β, can cross the BBB and initiate or modulate astrogliosis.

Peripheral and central immune cells express adrenergic and GCs receptors (Amsterdam, Tajima & Sasson, 2002; Marino & Cosentino, 2013), making them reactive to sympathetic signalling from the ANS and the HPA axis activation. GCs usually exert an anti‐inflammatory effect (Boumpas, Chrousos, Wilder, Cupps & Balow, 1993) and improve stress‐induced defence mechanisms (Munck & Náray‐Fejes‐Tóth, 1994). However, dysregulation of the loop connecting the ANS, the peripheral immune organs and the CNS shifts the immune system to a stress‐sensitive response (Frank et al., 2013). Following a 10‐day chronic social stress paradigm (see Box 1), mice who become susceptible to stress (depressive‐like) show significantly higher corticosterone and circulating inflammatory monocyte levels than resilient or control animals (Gururajan, Wouw, Boehme, Becker & O'Connor, 2019), suggesting a tight correlation between stress‐ and resilience‐related endocrine and immune dysregulation. In the next sections, we will review recent findings addressing this complex interaction between stress and immune responses.

### 3.3 Immune response in depression and resilience: insights from rodent studies

Psychological stress, like physiological challenges, can induce an increase in circulating monocytes, a process called monocytosis (Ginhoux & Jung, 2014) (see Figure 2). Although immune response to stress can be beneficial, an exuberant and prolonged inflammatory response can lead to deleterious effects. Interestingly, individual variability in the peripheral immune system function is associated with susceptibility or resilience to chronic social stress in mice (Hodes et al., 2014). A substantial body of evidence has shown that activation of the innate immune system driven by chronic stress exposure in rodents stimulates enhanced proliferation and egress of immature, pro‐inflammatory myeloid cells from the bone marrow into the bloodstream (Heidt, Sager, Courties, Dutta & Iwamoto, 2014; Powell, Sloan, et al., 2013; Powell, Tarr, and Sheridan, 2013) (see Figure 2). Several of these studies have examined the changes in immune cell reactivity in chronic social stress paradigms (See Box 1 for details on each model and Table 1 for behavioural outcomes), which has been shown to drive an increase of bone marrow‐derived monocyte and granulocyte progenitor cells and induce blood monocytosis and granulopoiesis (Avitsur & Sheridan, 2009; Engler, Bailey, Engler, & Sheridan, 2004; Powell, Sloan, et al., 2013; Powell, Tarr, and Sheridan, 2013). Mice subjected to psychosocial stress exhibit a shift in leucocyte transcriptional profile favouring production and subsequent release of immature, pro‐inflammatory Ly6C^high^ monocytes and Ly6C^intermediate^ granulocytes into circulation (Powell, Sloan, et al., 2013; Powell, Tarr, and Sheridan, 2013) (see Figure 2). This shift in transcriptional pattern occurs due to enhanced expression of pro‐inflammatory genes and is β‐adrenergic receptor signalling‐dependent (Powell, Sloan, et al., 2013; Powell, Tarr, and Sheridan, 2013). A recent study has found that chronic social stress causes a similar increase in levels of Ly6c^high^ monocytes in both resilient and susceptible mice, suggesting that intrinsic mechanisms within these immune cells could drive stress susceptibility vs resilience (Pfau, Menard, Cathomas, Desland & Kana, 2019). Similarly, mice subjected to chronic variable stress (CVS, see Box 1) have been reported to exhibit stress‐enhanced hematopoietic activity in blood and bone marrow, compared to home cage controls (Heidt et al., 2014). This stress‐induced increase in circulating neutrophils and Ly6c^high^ monocytes is also driven by sympathetic nervous system innervation of bone marrow via β‐adrenergic signalling (Heidt et al., 2014; McKim, Weber, et al., 2018; McKim, Yin, et al., 2018).

Interestingly, not only number but also reactivity of cells from the innate immune system is affected following chronic stress exposure. Mice subjected to psychosocial stress display exacerbated release of pro‐inflammatory cytokines TNF‐α and IL‐6 in response to treatment with bacteria‐derived endotoxin lipopolysaccharide (LPS) (Avitsur, Kavelaars, Heijnen & Sheridan, 2005; Hodes et al., 2014) (see Box 1). This effect is mediated by stress‐induced splenocyte GC resistance and varies between individuals based upon level of social subordination (Avitsur et al., 2005). Indeed, in comparison with unstressed controls and dominant mice, animals displaying submissive behaviours following a chronic social stress paradigm are more likely to develop splenocyte GC resistance (Avitsur et al., 2005). This indicates that adaptive mechanisms underlying GC downregulation of the immune system activation in response to stress could be present in dominant mice characterized by resilient behaviours, whereas impairment of these processes leads to submissive behaviours similar to those observed in stress‐susceptible mice following 10 days of social stress (see Box 1). Despite these promising data, our understanding of these effects of stress on the innate immune system is far from complete with more research needed to unravel the molecular mechanism as well as the immune profile of resilience.

Levels of circulating pro‐inflammatory cytokines, including IL‐1β and IL‐6, are elevated in rodent models of depression (Grippo, Francis, Beltz, Felder & Johnson, 2005; Hodes et al., 2014) (see Figure 2). Sustained and unresolved inflammation is a hallmark of chronic stress‐related pathologies (Maes, Stevens, et al., 1992; Maes, Van der Planken, et al., 1992). Animals receiving systemic administration of IL‐1β, TNF‐α or LPS show heightened expression of pro‐inflammatory cytokine genes and proteins in the brain (Layé, Parnet, Goujon, & Dantzer, 1994; Quan, Stern, Whiteside & Herkenham, 1999). Moreover, administration of those agents promoted development of sickness behaviours such as social withdrawal, loss of appetite, decreased motor activity and cognitive deficits in rodents (Anisman & Merali, 2002; Bonaccorso et al., 2001, 2002; Dantzer, O'Connor, Freund, Johnson & Kelley, 2008; Sakic, Gauldie, Denburg, & Szechtman, 2001) (see Box 1). Interestingly, resilient animals do not display exacerbated immune responses following acute or chronic stressors (see Figure 2). Furthermore, pre‐existing individual differences in the peripheral modulation of circulating leucocytes and their IL‐6 release following LPS stimulation predict stress susceptibility or resilience in chronic social stress in mice (Hodes et al., 2014). A single exposure to an aggressor is sufficient to significantly increase circulating levels of IL‐1β and IL‐6 in the blood of mice that subsequently become susceptible when compared to mice that will be considered resilient (Hodes et al., 2014). Results from chimeric mice subjected to transplantation of bone marrow‐derived hematopoietic stem cells from stress‐susceptible or IL‐6 knockout (IL‐6^−/−^) mice further highlighted a role for peripheral IL‐6 in the development of stress vulnerability. Indeed, stress‐susceptible bone marrow recipient chimeras display a robust social avoidance phenotype vs control bone marrow recipient chimera (Hodes et al., 2014). Conversely, chimeras generated via transplantation of progenitors from an IL‐6^−/ −^ donor exhibit resilience to chronic social stress. Similarly, systemic administration of IL‐6 monoclonal antibody, which binds and neutralizes IL‐6 in the peripheral circulation, enhances resilience in this stress paradigm (Hodes et al., 2014). These intriguing findings highlighting a role for peripheral IL‐6 in stress vulnerability vs resilience have been reinforced by a subsequent study reporting increased IL‐6 peripheral release in rats displaying learned helplessness (LH), a deficit in instrumental response to aversive events (Yang, Bertolucci, Wolf & Heisenberg, 2013) (see Box 1). Moreover, IL‐6 has been shown to induce a primed transcriptional profile in monocytes recruited to the brain associated with the development of anxiety following psychosocial stress in mice (Niraula, Witcher, Sheridan & Godbout, 2019). This was supported by the observation that anxious behaviours and social avoidance are prevented in IL‐6 knockout mice, despite stress‐induced monocyte release and recruitment to the brain (Niraula et al., 2019). Interestingly, positive changes in levels of IL‐6 release correlated negatively with social interaction ratio, indicating a predictive relationship. Based on these findings, pre‐existing differences in stress response leading to peripheral IL‐6 release could drive the adaptations responsible for development of stress susceptibility or resilience to chronic social stress.

It has recently been found that differences in leukocyte intrinsic processes, such as microRNA profile regulation, can contribute to the resilient phenotype (Pfau et al., 2019). The adaptive immune system plays a protective role by producing long‐lived memory T cells, building an appropriate adaptive immune response to future challenges (Batuman, Sajewski, Ottenweller, Pitman & Natelson, 1990; Miyajima, Zhang, Sugiura, Sonomura & Guerrini, 2017; Pfau et al., 2019; Slota & Weng, 2014) (see Figure 2). Psychological stress enhances T‐cell trafficking to the brain in mice, thereby providing a potential link between lymphocytes and adaptations to chronic stress (Cohen, Ziv, et al., 2006; Cohen, Doyle, & Baum 2006; Lewitus, Wilf‐Yarkoni, Ziv, Shabat‐Simon & Gersner, 2009). It is speculated that the adaptive immune system may also store immunological memory of a stressor and thus could protect an individual against similar future stress exposure (Lewitus & Schwartz, 2009; Lewitus et al., 2009; Rook & Lowry, 2008) (see Figure 2). Concomitantly, T‐cell‐dependent immunization with a CNS‐associated antigen, induced prior to exposure to chronic mild stress, prevents subsequent depression‐like behaviours in rats (Lewitus et al., 2009). Conversely, higher stress vulnerability has been shown in T‐cell‐deficient mice and injection of a single population of T cells reactive to CNS‐related antigens is sufficient to promote resilience (Cohen, Ziv, et al., 2006; Cohen, Doyle, et al., 2006). Altogether, these rodent preclinical studies indicate that appropriate adaptive immune system response prior to stress exposure may have a protective effect against stress‐related pathologies.

At the central level, chronic stress and systemic inflammation are known to activate microglia in rodents and alter their density and morphology, particularly in stress‐sensitive brain regions, such as the hippocampus, the prefrontal cortex and amygdala (Palin, Cunningham, Forse, Perry & Platt, 2008; Tynan, Naicker, Hinwood, Nalivaiko & Buller, 2010) (see Figure 2). Moreover, it has been shown that microglial activation is sustained after stress stimuli cessation. Indeed, prolonged microglia sensitization with a unique messenger RNA signature has been observed 24 days following psychosocial stress paradigm in mice (Weber, McKim, Niraula, Witcher & Yin, 2019). Peripherally derived pro‐inflammatory signals can reach the CNS either via the neural pathway including vagal nerves and brainstem nuclei stimulation or via the humoral pathway by crossing the BBB (Dantzer et al., 2008; Pavlov & Tracey, 2012; Quan, 2008; Wohleb, Fenn, et al., 2013; Wohleb, Powell, et al., 2013). Within the brain, both centrally derived inflammatory signals, produced by resident microglia, as well as infiltrating peripheral pro‐inflammatory cytokines can influence behaviour through activation of the HPA axis evoking subsequent GC signalling as well as excitatory synaptic plasticity (Boersma et al., 2011; Christoffel, Golden & Russo, 2011; Iwata, Ota & Duman, 2013). For example, one of the downstream mechanisms of stress‐induced IL‐1β release is modulation of the HPA axis followed by a downstream release of GCs (Berkenbosch, Oers, Rey, Tilders & Besedovsky, 1987; Iwata et al., 2013; Sapolsky, Rivier, Yamamoto, Plotsky & Vale, 1987). Among central stress‐induced inflammatory processes, a prominent role of microglial IL‐1β signalling has been revealed by numerous studies (Berkenbosch et al., 1987; Iwata et al., 2013; Sapolsky et al., 1987). Both acute and chronic stressors can activate the cytosolic pattern recognition receptor nucleotide‐binding domain and leucine‐rich repeat protein‐3 (NLRP3) inflammasome, constitutively expressed in microglia and macrophages, which in turn induces IL‐1β release in the brain (see Figure 2). Indeed, acute restraint stress (see Box 1) is sufficient to trigger the NLRP3 inflammasome pathway in the hippocampus, a brain region where the concentrations of microglia and IL‐1β receptors are the highest (Farrar, Kilian, Ruff, Hill & Pert, 1987). IL‐1β derived from activated microglia is associated with increased depression‐like behaviours in rodents (Han et al., 2019; Iwata, Ota, Li, Sakaue & Li, 2016; McKim, Weber, et al., 2018; McKim, Yin, et al., 2018). In fact, psychosocial stress induces microglia‐dependent recruitment of circulating IL‐1β‐producing monocytes in mice stimulating the expression of brain endothelial interleukin‐1 receptor type 1 (IL‐1R1) and promoting anxiety‐like behaviours (McKim, Weber, et al., 2018; McKim, Yin, et al., 2018). A recent study showed that IL‐1R1 is mainly expressed by endothelial, ependymal, choroid plexus cells and dentate gyrus neurons in the CNS. Interestingly, endothelial IL‐1R1 can mediate sickness behaviour, neurogenesis impairment and leucocyte entry in the CNS, while ventricular IL‐1R1 is involved in monocyte recruitment (Liu, Nemeth, McKim, Zhu & DiSabato, 2019). Astrocytes express low levels of IL‐1R1, and microglia or brain macrophage do not express it in physiological conditions. IL‐1 is responsible for stimulating endothelial cells induced activation of microglia via a contact‐independent signalling, contributing to neuroinflammation and altered behaviour (Liu et al., 2019). Furthermore, intracerebroventricular administration of IL‐1β increases anxiety‐like behaviours and leads to spatial memory deficits (Song, Horrobin & Leonard, 2006). In line with these findings, pharmacological or genetic inhibition of IL‐1β receptor prevents failure to escape in the LH paradigm (Maier & Watkins, 1995) and rescues anhedonia in rats exposed to chronic unpredictable stress (Koo & Duman, 2008) (see Box 1). Conversely, NLRP3 null mutant mice exhibit a resilient phenotype under chronic stress conditions, whereas reduction of microglial activity by administration of the inhibitor minocycline abolishes the pro‐ramifying effect of stress and reverses induction of depressive‐like behaviours (Iwata et al., 2016). Interestingly, ramifying effects of chronic unpredictable stress on microglial function have shown to be hindered by blocking CX3CL1 to fractalkine receptor 1 (CX3CR1)‐mediated neuron‐microglia communication (Milior, Lecours, Samson, Bisht & Poggini, 2016). This has been further confirmed in CX3CR1‐deficient mice which exhibit resilience to stress‐induced depressive‐like behaviour in chronic unpredictable stress and chronic despair paradigms (Hellwig, Brioschi, Dieni, Frings & Masuch, 2016; Rimmerman, Schottlender, Reshef, Dan‐Goor & Yirmiya, 2017). These results highlight IL‐1β signalling as an important mediator of behavioural susceptibility and resilience to chronic stress in rodents. Targets promoting lower microglia reactivity as well as reduced NLRP3 inflammasome activation could represent an interesting therapeutic avenue.

The role of astrocytes in inflammatory processes is well established as they actively respond to cytokine signalling (Pekny & Nilsson, 2005). As mentioned in the previous section, pro‐inflammatory cytokines, such as IL‐1β, TNF‐α, IFN‐γ and TGF‐β, can cross the BBB and initiate or modulate astrogliosis, suggesting that stress‐induced exacerbated activation of these glial cells may contribute to depression pathogenesis (see Figure 2). For instance, upon activation, astrocytes express and release high levels of CCL2 (Ransohoff & Tani, 1998). CCL2 acts on its receptor (CCR2) on peripheral immune cells, promoting extravasation and infiltration of monocytes into the brain (Ransohoff & Tani, 1998; Sica, Wang, Colotta, Dejana & Mantovani, 1990). This suggests that astrocytes may be actively involved in stress‐induced recruitment of peripheral monocytes (Ransohoff & Tani, 1998). Indeed, an in vitro study has demonstrated that astrocyte‐mediated CCL2 release is sufficient to induce monocyte transmigration in a co‐culture model of human BBB (Weiss & Bermanm, 1998). In line with these findings, inhibition of astrocyte activation and subsequent CCL2 release leads to dampening of peripheral Ly6C^high^ monocyte recruitment and infiltration into the brain (Zheng, Yang, Cao, Xie & Liu, 2016). Interestingly, these effects have been shown to improve depression‐like behaviours induced by either inflammation or chronic social stress in mice (Zheng et al., 2016). Further studies are necessary to elucidate the role astrocytes might play in stress‐induced recruitment of peripheral immune cells and establishment of MDD.

### 3.4 Immune response in depression and resilience: insights from human studies

The first report showing a link between depression and inflammation in humans was from hepatitis C patients who received chronic interferon‐α treatment. A subset of those patients developed psychiatric complications including depression (Conversano, Carmassi, Carlini, Casu & Gremigni, 2015; Renault, Hoofnagle, Park, Mullen & Peters, 1987). Then, in the early 1990s, Smith proposed ‘The macrophage theory of depression’. Volunteers who were given monokines, cytokines produced by monocytes and macrophages, developed symptoms similar to those observed in MDD patients. Moreover, it was argued that women experience higher rates of depression because oestrogen increases IL‐1 secretion by macrophages (Smith, 1991). Since then, great correlational evidence has shown that depressed patients have high levels of circulating pro‐inflammatory cytokines, such as TNF‐α, IL‐6 and IL‐1β (Dowlati et al., 2010; Maes, Stevens, et al., 1992; Maes, Van der Planken, et al., 1992). A recent meta‐analysis comprising 3212 MDD participants and 2798 healthy controls has shown that IL‐6, TNF‐α, soluble IL‐12 receptor, CCL2, IL‐13, IL‐18, IL‐12 and the soluble TNF receptor 2 are elevated in MDD individuals compared to healthy controls, whereas IFN‐γ levels are reduced (Köhler, Freitas, Maes, Andrade & Liu, 2017). Moreover, elevated levels of serum IL‐6 were found in cohorts of treatment‐resistant MDD patients (Hodes et al., 2014). A longitudinal cross‐lagged twin difference study found a bidirectional association between inflammation and depressive symptoms (Huang, Su, Goldberg, Miller & Levantsevych, 2019). Inflammation, measured by circulating IL‐6 levels, was positively correlated with future depressive symptoms, while depressive symptoms were predictive of future inflammation, as measured by blood CRP levels. These correlations are not influenced by genetic or environmental cofounding factors, such as smoking, education or physical activity, adding to the growing knowledge of causal pathways linking inflammation and depression (Huang et al., 2019). These results further specify a cytokine and chemokine immune signature of MDD and treatment‐resistant depression, although further studies are needed to elucidate the specific mechanisms involved.

MDD patients exhibit increased numbers of blood leukocytes, monocytes and neutrophils, positively correlated with the overall severity of the disorder (Maes, Stevens, et al., 1992; Maes, Van der Planken, et al., 1992). These findings were mirrored in subjects with low socioeconomic status (SES), considered a form of chronic stress. Low SES subjects have higher relative and absolute counts of monocytes in blood and displayed a transcriptional profile promoting pro‐inflammatory monocytes and β‐adrenergic induction of myelopoiesis (Powell, Sloan, et al., 2013; Powell, Tarr, and Sheridan, 2013). As previously discussed, in mice, CCL2 leads to monocyte recruitment and infiltration into the brain (Zheng et al., 2016). In MDD patients who committed suicide, CCL2 gene expression was upregulated in the dorsal anterior cingulate cortex (Torres‐Platas, Comeau, Rachalski, Bo & Cruceanu, 2014), suggesting increased peripheral immune cell recruitment to the brain of depressed subjects. Studies investigating the effect of antidepressant treatments on immune response in humans have been contradictory, some reporting a decrease in peripheral inflammation (Kubera, Lin, Kenis, Bosmans & Bockstaele, 2001; Mutlu, Gumuslu, Ulak, Celikyurt & Kokturk, 2012; Słuzewska, Rybakowski, Laciak, Mackiewicz & Sobieska, 1995), while others suggest an increase (Hannestad, DellaGioia & Bloch, 2011; Kubera, Kenis, Bosmans, Kajta & Basta‐Kaim, 2004; Munzer, Sack, Mergl, Schönherr & Petersein, 2013) or even no effect on inflammatory response (Jazayeri, Keshavarz, Tehrani‐Doost, Djalali & Hosseini, 2010). These data provide compelling evidence in humans linking interindividual differences in stress vulnerability and heightened immune response.

The study of resilience in humans originated in the 1970s, when a group of researchers investigated children's normal development despite exposure to significant adversity, such as war, poverty or maltreatment (Masten, 2001). However, resilience is a difficult concept to study in humans, due to its multidimensional aspect. To this day, human studies on resilience have been mainly correlational, typically measured in natural settings where behavioural and biological endpoints are hard to pinpoint. One of the strategies that has been linked to a greater capacity to handle stressful situations is the use of active coping (Southwick, Vythilingam & Charney, 2005). A recent study found that weekly 90‐minute group drumming sessions increase social resilience and decrease depressive symptoms, assessed by psychometric measures. These patients had a shift towards an anti‐inflammatory profile, with a significant decrease in blood TNF‐α level and an increase in circulating anti‐inflammatory IL‐4 level (Fancourt, Perkins, Ascenso, Carvalho & Steptoe, 2016). Other evidence also suggests that immune system response could contribute to the deleterious effect of stress and subsequently resilience. It was proposed that positive affect reflects ‘one's level of pleasurable engagement with the environment’, such as happiness, joy and enthusiasm (Clark, Watson & Leeka, 1989). On a cellular basis, a 2016 study showed that high‐arousal positive affect (e.g. excitement) decreases soluble tumour necrosis factor‐alpha receptor II level, a marker of TNF activity, in breast cancer survivors (Cohen, Ziv, et al., 2006; Cohen, Doyle, et al., 2006). In law students, it was reported that optimism is associated with improved mood, higher numbers of T helper cells and higher NK cytotoxicity (Segerstrom, Taylor, Kemeny & Fahey, 1998). These studies suggest that positive affect could be a resilience factor, by buffering the negative immune impact of stress. To our knowledge, no such research was conducted in MDD patients. Another important point to consider when studying stress susceptibility and resilience mechanisms is the nature and timing of stress exposures. Resilience is a highly dynamic process, shaped by genetics, sex, hormones, immunity and development (for review, see Hodes & Epperson, 2019). For example, girls who experienced adolescent abuse, a form of early‐life stress, are more likely to develop internalizing coping strategies later in life, predictive of higher risk for post‐traumatic stress disorder (Herringa, Birn, Ruttle, Burghy & Stodola, 2013). Although our understanding of immune responses in depression and resilience is growing, further research should aim to establish human immune and molecular signature of resilience and investigate its underlying mechanisms.

### 3.5 Sex differences in stress‐induced immune responses

There are marked sex differences in the healthy adult immune system, in part due to the expression of unique X and Y chromosome genes. The X chromosome contains the largest number of immune‐related genes of the human genome, while the Y chromosome contains genes that epigenetically regulate expression of immune cells (Case, Wall, Dragon, Saligrama & Krementsov, 2013). Some genes located on the X chromosome are resistant to X chromosome inactivation, such as CXC chemokine receptor 3 (CXCR3) and genes involved in T‐cell function, resulting in their overexpression in women (Qin, Rottman, Myers, Kassam & Weinblatt, 1998; Wang, Syrett, Kramer, Basu & Atchison, 2016). This particularity could contribute to improved humoral and cell‐mediated immune response to infection observed in women (Klein, Jedlicka & Pekosz, 2010; Wang et al., 2016) and suggests an important role for genetic sex differences in immune function and response. Gonadal hormones also influence the immune system in a sex‐dependent manner. At low doses, oestrogen can increase secretion of interleukins by DCs and increase specific antibody secretion by B cells, while high oestrogen levels and androgens are generally immunosuppressive (Neigh, Nemeth & Rowson, 2016; Trigunaite, Dimo & Jørgensen, 2015; Young, Wu, Burd, Friedman & Kaffenberger, 2014). Overall, women exhibit higher innate and immune response to antigenic stimulation than men, which could therefore underlie sex differences in the aetiology of MDD and resilience.

In rodents, males and females show sex‐specific peripheral immune profiles and adaptations to chronic stress (Rainville & Hodes, 2019; Rainville et al., 2018). For example, at baseline, males have higher Toll‐like receptor 4 expression on macrophages and neutrophils while females rather show enhanced IL‐10 production, macrophage activation and phagocytic capacity (Klein & Flanagan, 2016; Spitzer, 1999) (see Table 2). As for exposure to an immune challenge, females display higher neutrophils phagocytic capacity following LPS administration (Aomatsu, Kato, Kasahara & Kitagawa, 2013). The activity of NK cells also differs between sexes following 7 weeks of chronic mild stress (CMS, see Box 1), with female and male mice showing decreased and increased NK cell activity, respectively (Pitychoutis, Griva, et al., 2009; Pitychoutis, Nakamura, et al., 2009). Enhanced myelopoiesis, increased monocyte and granulocyte accumulation in the blood and spleen, and microglial hyperreactivity and monocyte recruitment in the brain were observed in both male and female mice subjected to psychosocial stress paradigm (McKim, Weber, et al., 2018; McKim, Yin, et al., 2018; Wohleb, Patterson, et al., 2014; Wohleb, McKim, et al., 2014; Yin, Gallagher, Sawicki, McKim & Godbout, 2019). These results highlight contrasting sex differences, but also similarities in adaptations to chronic stress in various animal models of depression.

**Figure 3:**
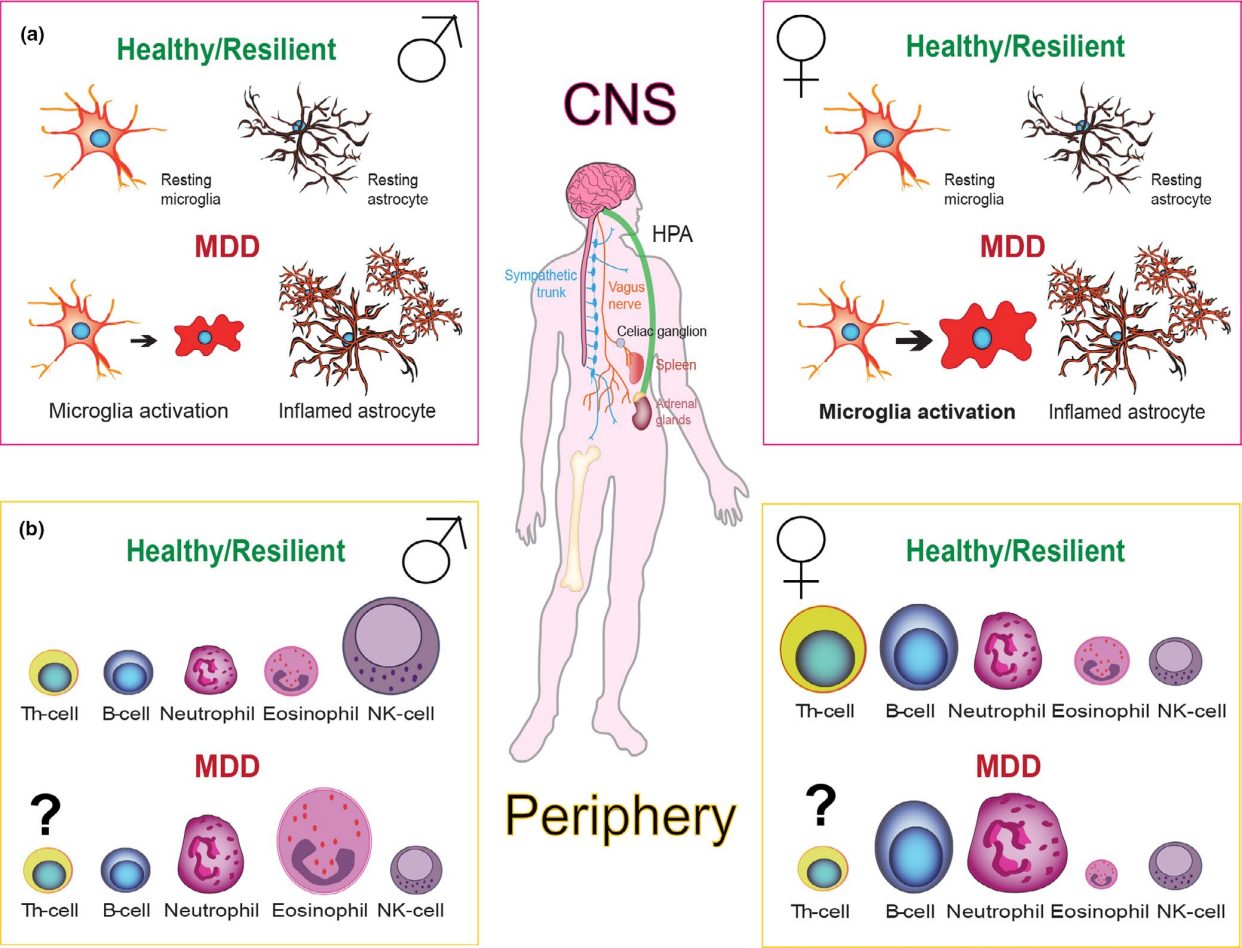
Differences in human immune system which could underlie sex differences in MDD pathogenesis. (a) In the central nervous system (CNS), the ratio of primed over ramified microglia is increased in MDD patients compared to healthy controls, with possibly exacerbated priming in MDD women. GFAP‐immunoreactive astrocyte levels and astrocyte packing density are found to be decreased in both male and female MDD subjects. (b) In the body periphery, healthy women have higher numbers of Th cells, B cells and neutrophils compared to healthy men. Both MDD men and women have increased neutrophils counts; however, this effect is more pronounced in women. Moreover, while healthy men have higher NK cell numbers, MDD men and women have similar numbers of circulating NK cells. Healthy individuals have low numbers of circulating eosinophils. However, stress results in increased eosinophil numbers in males, but decreased numbers in females. Regulation of T helper cells in MDD has been a matter of debate. Abbreviations: MDD: major depressive disorder; Th: T helper cell (CD4^+^); NK: natural killer cell

In humans, no difference in basal eosinophil number, morphology or structure was found between sexes (Sokol, James, Wales & Hudson, 1987). However, women have increased eosinophil reactivity, which may be explained by the presence of oestrogen receptor alpha (ERα) on their surface and higher circulating levels of oestradiol in women than in men (Keselman & Heller, 2015). On the other hand, men show marked higher basal NK cell activity as compared to women with regular menstrual cycles, although no significant difference in blood numbers of NK cell was found. Interestingly, women taking oral contraceptives have the lowest levels of NK activity, suggesting that hormone signalling might modulate NK cells activity (Yovel, Shakhar & Ben‐Eliyahu, 2001). Healthy women show higher percentages of B cells, T helper (CD4 + ) cells and higher T helper/T cytotoxic (CD8+) cell ratio than healthy men (Abdullah, Chai, Chong, Tohit & Ramasamy, 2012). However, conflicting data were reported in MDD patients, as some studies show an increased number of CD4+ cells (Maes, Stevens, et al., 1992), while others report an impaired maturation of two subtypes of T helper cells, Th2 and Th17 (Grosse, Hoogenboezem, Ambrée, Bellingrath & Jörgens, 2016) (see Figure 3).

As previously mentioned, increased levels of pro‐inflammatory cytokines, including IL‐6, IL‐1β and TNF‐α, were found in the blood of depressed patients (Pandey, Rizavi, Ren, Fareed & Hoppensteadt, 2012) in line with rodent findings. In both men and women, increased circulating levels of IL‐6 at 9 years old predicts greater likelihood of developing depression by the age 18 (Khandaker, Pearson, Zammit, Lewis & Jones, 2014). Interestingly, elevated serum levels of IL‐8, IFN‐γ and leptin were found in the blood of depressed women when compared to healthy controls, a difference that was not observed in men, suggesting that these pro‐inflammatory markers are sex‐specific in MDD patients (Birur, Amrock, Shelton & Li, 2017). Moreover, increased levels of IL‐6 and TNF‐α were reported in patients resistant to amitriptyline, a commonly prescribed antidepressant. However, patients with low blood IL‐6 and high TNF‐α levels responded well to this antidepressant treatment and showed reduced TNF‐α levels, concomitant with a 50% reduction levels in the Hamilton Depression Rating Scale (HAMD) and in the Montgomery and Åsberg Depression Rating Scale (MADRS), two clinical tools for MDD diagnosis (Lanquillon et al., 2000). These results suggest that individual differences in the peripheral immune system could be predictive of susceptibility to MDD or treatment success. Although beyond the scope of this review, the development and use of anti‐inflammatory drugs in clinical trials for MDD is rapidly growing, yielding interesting results (for detailed review, see Ménard, Pfau, Hodes & Russo, 2017)

As for central immune response, microglial number and morphology vary between sexes throughout brain development and regions. This phenomenon is attributed, in part, to fluctuation in levels of gonadal hormones during neonatal and postnatal development (Schwarz, Sholar & Bilbo, 2012). A recent study reported sex differences in the transcriptome of microglia from adult male and female mice with female microglia being characterized by a neuroprotective phenotype compared to males, independent from hormonal cues (Villa, Gelosa, Castiglioni, Cimino & Rizzi, 2018). At resting state, female rats have higher proportions of primed to ramified microglia in the prefrontal cortex and higher CX3CL1 to fractalkine receptor 1 (CX3CR1) signalling compared to males, suggesting higher inflammatory‐like state at baseline. However, following 10 days of chronic restraint stress (CRS; see Box 1), female rats have decreased proportions of primed microglia, an effect not observed in males, highlighting potentially more efficient anti‐inflammatory adaptations to chronic stress in females (Bollinger, Bergeon Burns & Wellman, 2016). Additionally, sex‐specific gene expression of pro‐ and anti‐inflammatory cytokines is present in microglia. In naive C57BL/6J mice, higher mRNA levels of TNF‐α, IL‐1β, IL‐6 and IL‐10 were found in females compared to males at postnatal day 3, suggesting that sex‐specific immune profiles are established early in development (Crain, Nikodemova & Watters, 2013) (see Table 2). Altogether, these animal data suggest that sex differences modulate differential microglial reaction to chronic stress, which could be involved in sexual dimorphism of mood disorders.

In humans, no difference in microglial density was observed between depressed patients and healthy controls (Schnieder, Trencevska, Rosoklija, Stankov & Mann, 2014; Steiner, Bielau, Brisch, Danos & Ullrich, 2008; Torres‐Platas, Cruceanu, Chen, Turecki & Mechawar, 2014). However, a study examining relative proportions of microglial phenotypes revealed increased ratio of primed over ramified microglia in the dorsal anterior cingulate cortex white matter of depressed individuals who committed suicide (Torres‐Platas, Cruceanu, et al., 2014) (see Figure 3). To date, very few studies have examined sex differences in microglial processes in MDD patients. A recent study performing large‐scale gene expression analysis across three corticolimbic brain regions found that genes specifically expressed in microglia were upregulated in men, but downregulated in women, with MDD (Seney, Huo, Cahill, French & Puralewski, 2018). These findings point towards distinct, maybe even opposite, molecular changes in MDD between sexes. Nevertheless, further studies are necessary to better understand sex‐specific differences of microglia in mood disorders and stress resilience.

Morphological and developmental sex differences are also present in astrocytes. Indeed, at basal state, higher number and processes’ complexity have been observed in astrocytes of the male rat medial amygdala when compared to females (Johnson, Breedlove & Jordan, 2008) (see Table 2). Gonadal hormones may play an important role in establishing those sex differences, as testosterone and oestradiol induce substantial changes in astrocyte processes and length (Amateau & McCarthy, 2002). In humans, several studies suggest that astrocytes are involved in MDD pathogenesis. Histopathological findings on postmortem brains report a decrease in packing density of Nissl‐stained glial cells in subjects with MDD (Gittins & Harrison, 2011; Ongur, Drevets & Price, 1998). These changes were observed in fronto‐limbic regions (Cotter et al., 2001, 2002; Rajkowska, Miguel‐Hidalgo, Wei, Dilley & Pittman, 1999) and in the amygdala (Bowley, Drevets, Öngür & Price, 2002), which are both involved in emotional regulation. Decreased GFAP‐immunoreactive astrocyte levels were also found in the amygdala (Altshuler et al., 2010), dorsolateral prefrontal cortex (Miguel‐Hidalgo, Baucom, Dilley, Overholser & Meltzer, 2000) and hippocampus (Cobb, O'Neill, Milner, Mahajan & Lawrence, 2016) of depressed patients as compared to healthy controls (see Figure 3). These later observations are in line with reported astrocytic loss in several limbic structures of a rat model of depression, including the prefrontal cortex and the basolateral amygdala (Gosselin, Gibney, O'Malley, Dinan & Cryan, 2009). Very few studies have investigated sex differences in astrocyte processes in the context of MDD. However, since astrocytes can produce and secrete pro‐inflammatory cytokines, including IL‐6, TNF‐α and IL‐1β (Farina et al., 2007; Lampron et al., 2013), which are mainly elevated in the blood of depressed women, they might play an important role in sexual dimorphism observed in MDD.

Several studies have highlighted a causal relationship between inflammation and depression both in mice and humans (Dowlati et al., 2010; Menard, Pfau, Hodes, Kana & Wang, 2017). However, the exact inflammatory mechanisms underlying depression and whether inflammation is a cause or consequence of depression are still unclear. Proper immune responses are vital and beneficial to some extent; however, exacerbated activation of the immune system is detrimental in several conditions. Understanding how the immune system works during the pathophysiology of depression is key to developing new pharmacotherapies for treatment‐resistant patients as well as for the discovery of biomarkers that could help with MDD diagnosis or other mood disorders. Human studies have reported contradictory results regarding the anti‐inflammatory effect of current antidepressants (Hannestad et al., 2011; Kubera et al., 2001; Munzer et al., 2013; Mutlu et al., 2012), reinforcing the need for further investigation addressing the potential antidepressant activity of anti‐inflammatory drugs or the efficacy of combined therapies for treating depression. A myriad of studies showed that depression induces biological changes in several CNS and peripheral cells which could contribute to the complexity of the disorder and its treatment. Increased number of immune cells concomitant with increased levels of pro‐inflammatory molecules is frequently observed in animal models of depression and in MDD patients (Cohen, Ziv, et al., 2006; Cohen, Doyle, et al., 2006; Dowlati et al., 2010; Grippo et al., 2005; Hodes et al., 2014; Pfau et al., 2019; Powell, Sloan, et al., 2013; Powell, Tarr, and Sheridan, 2013). For example, stress can induce monocytosis and granulopoiesis and increase egress of immature and pro‐inflammatory myeloid cells from the bone marrow to the blood (Engler et  al., 2004; Avitsur & Sheridan, 2009; Ginhoux & Jung, 2014; Powell, Sloan, et al., 2013; Powell, Tarr, and Sheridan, 2013). Interestingly, individual differences in the peripheral immune system can predict susceptibility or resilience to chronic social stress in mice (Hodes et al., 2014). These findings exalt the importance of establishing a fine‐tune immune profile of the changes associated with chronic stress and MDD. By exploring such immune differences, MDD vulnerability or resilience could become predictable, giving susceptible individuals an incentive to engage in pro‐coping activities in order to prevent the development of this disorder. It could even help design more appropriate personalized pharmacological therapies. In this regard, assessment of circulating cytokine levels represents an attractive target (for review, see Hodes, Kana, Menard, Merad & Russo, 2015). Pro‐inflammatory IL‐6 has gained increasing attention since its peripheral levels have been demonstrated to be consistently elevated in depressed humans (Dowlati et al., 2010; Hodes, Ménard & Russo, 2016). Higher IL‐6 circulating levels in childhood can predict greater risk for depression later in life (Khandaker et al., 2014), indicating that IL‐6 is a promising biomarker for diagnosing and predicting depression. However, longitudinal studies are necessary to confirm causal relationship when considering populations with distinct genetic backgrounds and immune signatures. Indeed, treatment with an IL‐6 monoclonal antibody was shown to be effective in promoting resilience in mice, raising the hypothesis that reducing peripheral pro‐inflammatory cytokine levels could help achieve remission of depressive symptoms in humans, at least in subpopulations of MDD patients characterized by exacerbated immune response (Hodes et al., 2014). Besides that, IL‐1β is another important mediator of stress susceptibility and increased circulating levels are also observed in MDD (Dowlati et al., 2010). Its production in the brain is related among others to the NLRP3 inflammasome, a protein complex mainly expressed by microglia and involved in stress response and depression (Iwata et al., 2013). Understanding how these cytokines communicate with the brain under stressful conditions and which are the most affected brain regions will help refine strategies to treat depression. Finally, important sex differences are present in the immune system which can be due to sex chromosome genes (Case et al., 2013) or gonadal hormones (Neigh et al., 2016; Trigunaite et al., 2015; Young et al., 2014). A distinct immune profile and variable levels of cytokines are observed at baseline and after stress in male vs female rodents (Rainville & Hodes, 2019; Rainville et al., 2018). Studies in humans corroborate these findings with sex‐specific changes in the immune profile of depressed subjects (Birur et al., 2017). Deciphering sex differences in stress‐induced immune responses could provide important insights into the dimorphic mechanisms underlying stress, depression and resilience. Moreover, it might help to decrease the low responsiveness of some patients to current pharmacological treatments that, in part, might occur since few studies addressed biological sex differences in the past. Overall, despite the great body of evidence associating immune responses and depression, there is still a lot to discover about the pathways linking them together. A better understanding of such connections will contribute to the discovery of new therapeutic targets allowing the development of novel, hopefully more effective MDD treatments.

## 4 VASCULAR FUNCTION IN DEPRESSION AND RESILIENCE TO STRESS

### 4.1 Overview of the blood–brain barrier and neurovasculature

Despite a significant body of evidence showing an association between activated immune system and susceptibility to stress, a question remains: How can stress ‘enter the brain’ to affect immune response? The BBB is an important, dynamic interface between the brain parenchyma and the systemic circulation (Abbott, Patabendige, Dolman, Yusof & Begley, 2010). Under homeostatic conditions, the BBB tightly controls communication and transport of material to and from the brain. The BBB is part of the neurovascular unit (NVU) that consists of numerous cell types, including endothelial cells, pericytes, astrocytes, microglia and neurons. The activity of different cell types of the NVU is tuned together to ensure evenness and efficiency of cerebral blood supply, which can satisfy the rapid changes in metabolic demand ensuing due to the neuronal activation (Muoio, Persson & Sendeski, 2014). The BBB is vital in regulating these functions due to its ability to control the exchange of ions and nutrients between the blood and the brain (Abbott et al., 2010). BBB properties are important for protection of CNS homeostasis as it serves as the brain's first line of defence preventing potentially harmful signals, such as pathogens, immune cells and anaphylatoxins circulating in the blood from entering into the brain (Abbott et al., 2010). BBB properties as the interface between the brain and the peripheral vasculature are attained by highly electrical‐resistant tight junction proteins, together with polarized transporter proteins and other surrounding NVU cells limiting the molecule transport to the cerebral parenchyma.

Endothelial cells of the cerebral microvasculature have a unique phenotype, with increased density of mitochondria and characteristic polarization in the expression of specific transporters and junctional complexes that regulate the paracellular transport of molecules and ions to the brain (Daneman, 2012; Nag, 2011). The interendothelial space of the cerebral microvasculature is characterized by the presence of ‘kissing points’, where tight junction proteins of adjacent endothelial cells interact to seal the paracellular space (Keaney & Campbell, 2015). The binding of tight junction proteins impedes the flow of solutes and ions bidirectionally between peripheral blood supply and the cerebral parenchyma. Additionally, this maintains polarity by enabling asymmetric distribution of membrane constituents, creating a dynamic and highly versatile barrier system (Tsukita, Furuse & Itoh, 2001). Major components of tight junctions are claudin and occludin integral membrane proteins with a structure that consists of four transmembrane domains and two extracellular loops (Vorbrodt & Dobrogowska, 2003). Claudins and occludins are linked to the actin cytoskeleton by zonula occludens (ZO) complexes on the intracellular domain of the plasma membrane (Fanning, Jameson, Jesaitis & Anderson, 1998; Hartsock & Nelson, 2008).

Structurally, brain endothelial cells are in contact with astrocytic terminal processes, known as end‐feet and pericytes through the cellular basal lamina, which is crucial to maintain BBB function and integrity (Hawkins & Davis, 2005; Najjar, Pearlman, Alper, Najjar & Devinsky, 2013; Stanimirovic & Friedman, 2012). Astrocytic end‐feet establish the link between the endothelial cells and neurons enabling the modulation of both neuronal activity and cerebral blood flow, in response to an increase in Ca^2+^ signalling (Maragakis & Rothstein, 2006; Zonta, Angulo, Gobbo, Rosengarten & Hossmann, 2003). Indeed, astrocytic end‐feet express specialized molecules such as Kir4.1 K^+^ channels and aquaporin 4 (aqp4) that regulate BBB ionic concentrations (Alvarez, Katayama & Prat, 2013). Additionally, well‐coordinated astrocytic calcium signalling communication via gap junctions regulates vasodilation and vasoconstriction of BBB vasculature (Alvarez et al., 2013; Theis, Söhl, Eiberger & Willecke, 2005).

Pericytes are located in between the endothelial cells, astrocytic end‐feet and neurons, embedded in the basal membrane and enwrapping cells of blood microvessels (Wong, Ye, Levy, Rothstein & Bergles, 2013). They are responsible for the maintenance of BBB homeostasis by regulating permeability, angiogenesis, cerebral blood flow and clearance (Dore‐Duffy & Cleary, 2011). Indeed, pericytes have been shown to regulate the expression of BBB tight junction proteins, such as claudin‐5 (cldn5) (Bell, Winkler, Sagare, Singh & Larue, 2010; Shimizu,, Sano, Abe, Maeda & Ohtsuki, 2011). Moreover, these cells display some macrophage‐like features with expression of markers such as CR3 complement receptor and class I and II major histocompability complex molecules as well as scavenger receptors (Balabanov, Washington, Wagnerova & Dore‐Duffy, 1996; Pieper, Marek, Unterberg, Schwerdtle & Galla, 2014). Homeostatic function of the BBB has been shown to be challenged under chronic stress condition in humans and rodents (Friedman, Kaufer, Shemer, Hendler & Soreq, 1996; Niklasson & Ågren, 1984) as will be discussed in the next sections. Additionally, increasing evidence suggests that stress‐induced perturbation of BBB functions may be involved in the pathophysiology of MDD. Therefore, characterizing the components as well as the mechanisms maintaining a healthy and hypopermeable BBB is a promising strategy for preventing the CNS from damage and disease.

### 4.2 Regulation of the blood–brain barrier in stress susceptibility and resilience

In recent decades, multiple animal studies have suggested potential BBB dysfunction in response to acute and chronic stress (Cheng, Desse, Martinez, Worthen & Jope, 2018; Esposito, Gheorghe, Kandere, Pang & Connolly, 2001; Menard et al., 2017; Pearson‐Leary, Eacret, Chen, Takano & Nicholas, 2017; Sántha et al., 2016; Sharma & Dey, 1981; Xu, Li, Ma, Wang & Sun, 2019; Zhao et al., 2017). While most focused solely on stress‐induced depressive behaviours (see Table 1), recent preclinical studies including rodent models of stress‐resilient subpopulations (see Box 1) have provided novel insights on how chronic stress disrupts BBB function leading to depression or stress resilience (Cheng et al., 2018; Menard et al., 2017). Menard *et al*. showed that expression of endothelial tight junction protein cldn5 is reduced in the nucleus accumbens (NAc) of stress‐susceptible vs resilient mice or unstressed controls allowing passage of circulating pro‐inflammatory IL‐6 into the brain (Menard et al., 2017). The NAc is a brain region crucial for regulation of motivated behaviour, and its function is impaired in both MDD patients and animals exposed to chronic stress (Menard et al., 2017; Satterthwaite, Kable, Vandekar, Katchmar & Bassett, 2015). Both mRNA and protein levels of cldn5 in the NAc correlated with social behaviours, suggesting that loss of tight junctions and BBB integrity contributes to stress vulnerability. In fact, stress‐induced or viral‐mediated increase in BBB permeability led to infiltration of Evans blue, a dye with high affinity for blood serum albumin, in the NAc of stress‐susceptible mice (Menard et al., 2017). BBB leakiness was further validated through magnetic resonance imaging (MRI) scans revealing higher infiltration of gadolinium contrasting agent in the NAc of stress‐susceptible mice when compared to unstressed controls and resilient mice (Menard et al., 2017). Of note, reduced CLDN5 expression was also detected in postmortem NAc samples of MDD subjects who committed suicide, confirming that neurovascular dysfunction is also present in human depression (Menard et al., 2017). Interestingly, chronic treatment with the antidepressant imipramine was sufficient to prevent the stress‐induced decrease in clnd5 expression, in line with restoring normal social behaviour in mice (Menard et al., 2017). However, further studies are required to better understand how antidepressant treatment affects stress‐induced vascular dysfunction.

Another study using a chronic social stress paradigm but conducted in rats found that animals characterized by passive coping mechanisms, which are associated with vulnerability to stress, display heightened vascular remodelling, including an increase in BBB permeability in the hippocampus (Pearson‐Leary et al., 2017). Vascular dysfunction was not observed in actively coping animals displaying stress resiliency (Pearson‐Leary et al., 2017) in line with Menard et al. (2017). Increased BBB permeability in the mouse hippocampus was also observed following the LH paradigm (Cheng et al., 2018) (see Box 1). This effect is maintained in mice with prolonged LH, which is an equivalent of inadequate stress coping mechanisms, despite the fact that BBB integrity was normalized after LH recovery (Cheng et al., 2018). Interestingly, administration of a TNF‐α inhibitor in non‐recovered animals reversed BBB hyperpermeability, promoting recuperation (Cheng et al., 2018).

As mentioned previously, susceptibility to chronic social stress is characterized by exacerbated peripheral and central inflammatory responses which represent an attractive mechanism to explain high comorbidity between inflammatory conditions such as cardiovascular diseases and depression (Finnell & Wood, 2016; Miller, Stetler, Carney, Freedland & Banks, 2002). Indeed, impairment of endothelium‐dependent vasorelaxation indicating vascular dysfunction has been shown in the chronic unpredictable mild stress model of depression (Isingrini, Surget, Belzung, Freslon & Frisbee, 2011). Repeated stress exposure could affect endothelial cells of the neurovasculature promoting an inflammatory profile and facilitating propagation of circulating neuroinflammation into the CNS. Accordingly, Menard et al. (2017) showed that loss of BBB integrity resulted in passage of the pro‐inflammatory cytokine IL‐6 into the mouse NAc leading to social avoidance. Peripheral IL‐6 is necessary for development of maladaptive synaptic plasticity in the NAc of susceptible mice following psychosocial stress (Wang, Hodes, Zhang, Zhang & Zhao, 2018), suggesting that BBB leakiness could actively participate in depression pathogenesis. As mentioned in the previous section, meta‐analysis studies of MDD patient population have confirmed elevated serum levels of pro‐inflammatory cytokines, notably TNF‐α and IL‐6 (Liu, Ho & Mak, 2012; Maes, 1995; Miller, Maletic & Raison, 2009). Negative effects of pro‐inflammatory cytokines on BBB permeability have been demonstrated both in animal (Banks, Kastin, & Gutierrez, 1994; Banks, Kastin, & Broadwell, 1995; Henninger, Panés, Eppihimer, Russell & Gerritsen, 1997; Van Dyken & Lacoste, 2018; Zameer & Hoffman, 2003) and in human subjects (Becker, Quay & Soukup, 1991; Haraldsen, Kvale, Lien, Farstad, & Brandtzaeg, 1996; Li, Paul, Ko, Sheldon & Rich, 2012).

Loss of BBB integrity can be mediated by intercellular adhesion molecule‐1 (ICAM‐1) expression and subsequent leukocyte binding and transmigration into the luminal surface of BBB endothelial cells (Dietrich, 2002; Haraldsen et al., 1996; Henninger et al., 1997). Abrogation of corticosterone signalling during a psychosocial stress paradigm has been shown to attenuate neurovascular expression of ICAM‐1 (Niraula, Wang, Godbout & Sheridan, 2018). Indeed, mice subjected to social stress present increased endothelial expression of ICAM‐1 and vascular cell adhesion molecule 1 (VCAM‐1) in the hippocampus and amygdala which promotes peripheral myeloid cell trafficking to the brain, contributing to behaviour impairment (Sawicki, McKim, Wohleb, Jarrett & Reader, 2015). Once leukocytes are activated by chemokines, their conformation changes allowing firm adhesion to the surface of the endothelium through integrin binding. VCAM‐1 and ICAM‐1 are both involved in this process preceding extravasation of peripheral leucocytes through the vascular wall (for review, see Vestweber, 2015). These two integrin ligands are expressed in both large and small blood vessels after stimulation of endothelial cells by cytokines and form an actin‐supported platform enhancing leukocyte‐endothelial cell binding. These interactions can stimulate actomyosin filament‐mediated contractions leading to tight junction opening and passage of peripheral immune cell into the brain activating a central immune response. Corticosterone depletion reduces inflammatory response by decreasing stress‐induced microglial remodelling but also by preventing monocyte accumulation in the brain and neuroinflammatory signalling (Niraula et al., 2018). Despite evidence that chronic stress promotes a reactive endothelium in a brain region‐dependent manner and differentially affects the BBB in stress‐susceptible vs resilient animals, cellular mechanisms or molecular changes driving these biological differences are still unknown.

Astrocytes can regulate expression of endothelial tight junction proteins occludin, cldn5 and ZO‐1 when co‐cultured with primary endothelial cells (Argaw et al., 2009, 2012; Kröll, El‐Gindi, Thanabalasundaram, Panpumthong & Schrot, 2009). Further arguments suggesting astrocyte involvement in regulating BBB permeability are derived from studies of the astroglial end‐feet process water channel, aqp4 expression. Indeed, it has been proposed that decreased density of the aqp4 expression may impair glial‐vascular communication, critical for NVU homeostasis, and can lead to an increase in BBB permeability (Nicchia, Nico, Camassa, Mola & Loh, 2004). Further support for the involvement of astrocyte‐related mechanisms in BBB hyperpermeability comes from studies showing astroglial loss and reduction of astrocyte‐specific markers, such as GFAP in rodent models of chronic stress in regions involved in stress response (Nagy, Suderman, Yang, Szyf & Mechawar, 2015; Torres‐Platas, Nagy, Wakid, Turecki & Mechawar, 2016; Tynan, Beynon, Hinwood, Johnson & Nilsson, 2013). Chronic, pathological activation of astrocytes eventually leading to apoptosis might negatively affect BBB permeability. Additionally, treatment with cotinine, an alkaloid found in tobacco with effects on motivation and cognition, during restraint stress has been shown to prevent both stress‐induced depression‐like behaviours and changes in number and arborization of GFAP+ cells in mice (Perez‐Urrutia, Mendoza, Alvarez‐Ricartes, Oliveros‐Matus & Echeverria, 2017). These protective actions have been observed in the prefrontal cortex and hippocampus, two brain regions highly involved in resilience to chronic stress conditions (Perez‐Urrutia et al., 2017). Moreover, astrocytes could contribute to stress resilience by regulating glutamate homeostasis in the ventral hippocampus of mice (Nasca, Bigio, Zelli, Angelis & Lau, 2017). Indeed, increased action of the astroglial glutamate exchanger, xCT, in the hippocampus promotes pro‐resilient and antidepressant‐like responses (Nasca et al., 2017). In agreement, xCT expression is reduced in a genetic mouse model with inherent susceptibility to depressive‐like behaviour (Nasca et al., 2017). Although astrocyte‐neuronal communication has been receiving greater attention recently (Murphy‐Royal, Gordon & Bains, 2019), to date, only a handful of papers have explored the involvement of astrocytes in stress resilience and future studies are greatly needed.

Microglia remain in constant bidirectional communication with endothelial cells to exert their surveying functions on the integrity of the BBB. Indeed, numerous studies have demonstrated a very tight spatiotemporal correlation between vascular activation, breakdown of the BBB and activation of brain‐resident microglia (Barkauskas, Dixon Dorand, Myers, Evans & Barkauskas, 2015; Neumann, Riek‐Burchardt, Herz, Doeppner & König, 2015). Microglia are highly reactive to psychological stress, with an increased number of activated microglial cells in limbic brain regions in animals subjected to chronic stress (Tynan et al., 2010; Wohleb et al., 2012). Similarly, mice subjected to psychosocial stress paradigm have been shown to have an increased neuroinflammatory response with higher presence of ramified Iba1 + microglia in the medial amygdala, prefrontal cortex and hippocampus (Wohleb et al., 2012, Wohleb, Powell, et al., 2013). Indeed, development of stress‐induced anxiety depended on monocyte IL‐1β production and stimulation of IL‐1R1 at the blood–brain interface (McKim, Weber, et al., 2018; McKim, Yin, et al., 2018). Notably, both monocyte recruitment and increased brain endothelial IL‐1R1 expression depended on pro‐inflammatory microglial activation (McKim, Weber, et al., 2018; McKim, Yin, et al., 2018). In agreement, endothelial IL‐1R1 knockdown mice did not develop anxiety following RSD stress paradigm (Wohelb et al., 2014). These data are underscored by findings from in vitro studies where LPS‐induced microglial activation decreased transendothelial electrical resistance of an endothelial monolayer by disrupting tight junction proteins, including cldn5 (Sumi, Nishioku, Takata, Matsumoto & Watanabe, 2010). Interestingly, blood pressure and angiotensin II, a peptide hormone involved in the development of hypertension, have been shown to contribute to hippocampal microglia activation in mice (Iulita, Vallerand, Beauvillier, Haupert & Ulysse, 2018).

In rodents, chronic social stress can affect the neurovasculature leading to depression‐like behaviours but also promote cardiovascular deficits including increased heart rate, blood pressure and arrhythmias (Sgoifo et al., 1999, 2014). In fact, repeated exposure to social stress has been shown to differentially contribute to depression‐like behaviours and comorbid vascular pathology based on physical versus purely psychological stressor involvement in the modified rat resident–intruder paradigm (Finnell, Lombard, Padi, Moffitt & Wilson, 2017). In this set of experiments, combined physical and psychological components of social stress were modelled as described in Box 1. In parallel, the psychological component of social stress was isolated by subjecting rats to a witness paradigm in which the animal only observed an intruder rat being defeated without ever being itself exposed to an aggressor (Finnell et al., 2017). This highlighted differences in prolonged effects of these stressors with psychological stress in the witness paradigm more likely to produce long‐term cardiovascular dysfunction and comorbid emergence of depressive‐like anhedonia. On the other hand, physiological and physical stressors combined as experienced in a chronic social stress paradigm primed sensitivity to inflammation (Finnell et al., 2017). Interestingly, blockade of CRF signalling promotes resilience with increased latency to submit to the resident in the rat resident–intruder paradigm (Wood, McFadden, Grigoriadis, Bhatnagar & Valentino, 2012). Additionally, aberration of CRF signalling reduces social stress‐induced ACTH and corticosterone release and decreased heart rate variability (Wood et al., 2012). Stress‐induced changes in cardiovascular function reinforce the need to consider peripheral vascular adaptations alongside assessment of BBB integrity to better understand how chronic stress can lead to depression or reveal mechanisms associated with resilience.

Despite an increasing amount of evidence indicating that stress influences the immune and vascular systems resulting in depression‐associated behavioural changes, specific mechanisms are still not well understood. It has been suggested that chronic stress induces neurovascular pathology, leading to BBB hyperpermeability which may in turn increase crosstalk between innate and adaptive immunity, thereby resulting in propagation of a neuroinflammatory response in the brain and MDD pathology.

### 4.3 Neurovascular adaptations in human depression

A link between neurovascular dysfunction and MDD pathology is supported by studies assessing peripheral vascular endothelial dysfunction in patients with depressive symptoms. For instance, MDD is associated with greater impairments in vascular conductance as measured by endothelium‐dependent dilatation (Greaney, Koffer, Saunders, Almeida & Alexander, 2019). Furthermore, a prospective study of subjects with various depressive disorders has shown a lower relative uptake ratio (RUR) of blood flow in the brachial artery of MDD patients in comparison with non‐depressed controls (Lavoie, Pelletier, Arsenault, Dupuis & Bacon, 2010). RUR is a measure done via nuclear imaging that evaluates vascular dilatory response, with a lower RUR implying poorer vascular endothelial function. The RUR differences observed in MDD patients remained statistically significant even after applying adjustments for factors like age, sex and comorbidity with other diseases (Lavoie et al., 2010). Similarly, pro‐apoptotic activity of human endothelial cells is increased in MDD patients compared to non‐depressed controls (Politi, Brondino & Emanuele, 2008). Once again, these effects remained statistically significant even after correcting for age and cardiovascular comorbidity (Politi et al., 2008).

The focal point of clinical studies linking increased BBB permeability with MDD pathophysiology has been assessment of differences in cerebrospinal fluid (CSF)‐to‐serum ratios of various molecules, such as albumin. Indeed, elevation of CSF‐to‐serum albumin ratio in a substantial subpopulation of MDD patients clearly supports BBB and/or blood–CSF barriers hyperpermeability (Bechter, Reiber, Herzog, Fuchs & Tumani, 2010; Gudmundsson, Skoog, Waern, Blennow & Palsson, 2007). Further support comes from a cross‐sectional study of elderly women that identified increased CSF to serum levels of peripheral markers including albumin and urate in MDD patients relative to non‐depressed controls (Gudmundsson et al., 2007). Increased CSF‐to‐serum albumin quotient ratio, a most reliable biomarker for estimating the BBB permeability as albumin originates only from the blood, was associated with abnormal slowing of the electroencephalogram, indicating cerebral dysfunction and suicidality (Niklasson & Ågren, 1984). Another biomarker of which increased serum levels are suspected to be an indicator of BBB dysfunction in depressed individuals is calcium‐binding protein S100β, a marker of glial activation. It is important to note that S100β levels have been shown to decrease following antidepressant treatment (Schroeter, Abdul‐Khaliq, Diefenbacher & Blasig, 2002). Moreover, the effect of antidepressant treatment on S100β levels was positively associated with clinical improvement in MDD patients (Ambrée, Bergink, Grosse, Alferink & Drexhage, 2015; Schroeter et al., 2002). Underscoring these data were a comparison of coronary artery bypass grafting procedures, where changes in levels of S100β positively correlated with remission of depressive symptoms (Pearlman, Brown, MacKenzie, Hernandez & Najjar, 2014).

The level of astrocyte‐related aqp4 is reduced in orbitofrontal cortical grey matter of individuals with MDD (Rajkowska & Stockmeier, 2013). Decrease in aqp4 density is suspected to contribute to increased cerebral perfusion and metabolic abnormalities as detected by positron emission tomography imaging in patients with MDD (Serlin, Levy & Shalev, 2011). These findings were further confirmed by studies exploring depression‐linked decrease in astrocyte density based on reduction in GFAP protein expression, a well‐established biomarker for mature astrocytes. Indeed, a substantial loss of total astrocyte volume and decrease in GFAP expression in the brains of patients with MDD have been observed by several groups (Cobb et al., 2016; Miguel‐Hidalgo et al., 2000; Rajkowska & Stockmeier, 2013). Depressive behaviours can be induced by pharmacologic astrocytic ablation with L‐alpha‐aminoadipic acid in the prefrontal cortex of rat (Banasr & Duman, 2008), further reinforcing interest in exploring if these glial cells could play a central role in stress responses and be manipulated to promote resilience.

In parallel, postmortem analysis of brain tissue from depressed patients who committed suicide suggests an increase in microglia activation (Steiner et al. 2011; Steiner et al., 2006, 2008). Activated microglia—positive for Iba‐1 marker—were observed within or in contact with blood vessel walls in dorsal prefrontal white matter in a suicide group (Schnieder et al., 2014). In support of this observation, a recent PET study in humans shows that there is greater microglial activation in cortical areas that directly correlate with depression severity (Holmes, Hinz, Conen, Gregory & Matthews, 2018). Importantly, reduction in glial density has been consistently documented in MDD‐associated human brain areas, such as prefrontal and cingulate cortices, amygdala and hippocampus (Cotter et al., 2001, 2002, 2005; Rajkowska & Stockmeier, 2013). On the other hand, a postmortem study reported decreased inflammatory markers in the choroid plexus of suicide subjects (Devorak, Torres‐Platas, Davoli, Prud'homme & Turecki, 2015). The choroid plexus is a highly vascularized tissue responsible for producing cerebrospinal fluid and presenting an important role in the interface between peripheral and CNS inflammation. Decreased inflammation in this tissue might be a compensatory mechanism conducted through its extensive network of frenestrated capillaires that attenuates peripheral inflammatory impact on the CNS (Devorak et al., 2015). These clinical findings suggest that neurovascular dysfunction, including abnormal changes of BBB permeability driven by exacerbated microglial activation, could contribute to MDD pathophysiology. Elucidating the differences in the vascular system components and the BBB among specific areas from the CNS will clarify their involvement in depression; however, more studies are needed in order to better elucidate the involvement of the neurovascular system in the pathophysiology of MDD.

### 4.4 Sex specificity in stress‐induced neurovascular adaptations

In both physiological and pathological conditions, sex‐specific distinctions exist in neurovasculature adaptations and functions, and these effects are largely driven by sex hormones. While they are mainly produced in the gonads, sex hormones can also be produced by extragonadal sites, such as the vasculature and the brain, where they act locally in a paracrine or autocrine manner (Simpson, Rubin, Clyne, Robertson & O'Donnell, 2000). In mice, oestradiol increases transendothelial resistance *in vitro*, as measured by increased cldn5 protein and mRNA levels, indicative of a tighter and less permeable BBB (Burek, Arias‐Loza, Roewer & Förster, 2010) (see Table 2). In male mice, chronic depletion of testosterone increases the permeability of the BBB, while testosterone replacement reverses these effects (Atallah, Mhaouty‐Kodja & Grange‐Messent, 2017). Moreover, gonadal steroid receptors, such as ERα, ERβ and adrenergic receptors, are expressed in the endothelial cell layer of the BBB of both rodents and humans (Zuloaga, Swift, Gonzales, Wu & Handa, 2012). Following 8 weeks of chronic unpredictable mild stress, more severe depression‐like behaviours were observed in female vs male mice in line with higher plasma cortisol (Stanley, Brooks, Butcher, d'Audiffret & Frisbee, 2014). Surprisingly, females were characterized by blunted vasculature impairment and decreased systemic inflammation when compared to males (Stanley et al., 2014), suggesting that sex‐related hormones affect vascular and immune responses underlying depressive behaviours. Sex‐specific expression levels of these hormones and their receptors in the neurovasculature are mostly unknown, although their presence suggests a substantial influence of sex steroids on neurovascular function in both sexes.

As previously mentioned, CSF‐to‐serum albumin quotient ratio is a reliable biomarker to estimate BBB permeability. A recent study conducted in more than 20 000 patients with various health conditions and 335 healthy volunteers has shown higher CSF‐to‐plasma albumin ratio in females than males, underlying sex differences in BBB integrity (Parrado‐Fernández, Blennow, Hansson, Leoni & Cedazo‐Minguez, 2018). To our knowledge, it is unknown whether sex differences are present for CSF‐to‐serum ratio in MDD patients. In a traumatic brain injury model in rats, although BBB alterations and microglial activation were similar across sexes at 1‐day post‐injury, female rats showed higher astrocytic hypertrophy whereas males presented increased endothelial activation and expression of β‐catenin, indicative of angiogenesis (Jullienne, Salehi, Affeldt, Baghchechi & Haddad, 2018). These results suggest that neurovascular adaptations to BBB insult may be sex‐specific and drive sexually dimorphic responses to physical and psychological stress.

Cerebral blood flow is also differentially regulated between males and females across lifespan. In childhood, cerebral blood flow is 9–15% higher in girls than in boys (Tontisirin, Muangman, Suz, Pihoker & Fisk, 2007), while in adulthood, it remains about 11% higher in women than men across all ages (Rodriguez, Warkentin, Risberg & Rosadini, 1988). As aforementioned, maintenance of cerebral blood flow is highly dependent on vasodilatation and vasoconstriction of the barrier, which is tightly regulated by secretion of vasoactive factors by endothelial cells. During adolescence and adulthood, women show higher autoregulation of cerebral blood flow than men (Deegan, Sorond, Galica, Lipsitz & O'Laighin, 2011; Vavilala, Kincaid, Muangman, Suz & Rozet, 2005), suggesting that it may be controlled by female sex hormones at least in humans (Brackley, Ramsay, Broughton Pipkin & Rubin, 1999; Diomedi, Cupini, Rizzato, Ferrante & Giacomini, 2001). Despite these major differences, little is known about sex‐specific neurovascular adaptations in chronic stress and MDD. Further studies are necessary to provide a better understanding of those differences and similarities, particularly in the context of stress resilience. We think that it could greatly contribute to identifying mechanistically dimorphic mechanisms and lead to the discovery of new therapeutic targets and development of novel and effective MDD treatments across sexes.

Preclinical and clinical studies have been suggesting that BBB permeability is altered in human depression and stressed rodents for decades (Friedman et al., 1996; Niklasson & Ågren, 1984; Sharma & Dey, 1981). Nevertheless, the cellular and molecular mechanisms underlying stress‐induced BBB hyperpermeability remain elusive. This could part be due to the lack of efficient tools such as endothelial cell‐specific viral vectors allowing manipulation of relevant gene expression before behavioural assessment or in vivo imaging prohibitive costs. Mounting evidence highlighting the importance of the immune system in stress responses and human depression (Hodes et al., 2015; Hodes et al., 2014; Menard et al., 2017; Miller & Raison, 2016; Wohleb, McKim, Sheridan & Godbout, 2015) could also have contributed to increasing interest in the role of the BBB in stress‐induced biological responses. Indeed, the BBB represents the ultimate frontier between deleterious circulating immune signals and the brain. Recent studies from different groups linking inflammation with BBB permeability in stress‐related brain regions (Cheng et al., 2018; Menard et al., 2017; Pearson‐Leary et al., 2017) provide a novel framework in which elevated levels of circulating cytokines, a hallmark of treatment resistance in subpopulations of depressed patients (Hodes et al., 2015; Menard et al., 2017), weaken the neurovasculature leading to passage of peripheral immune signals and contributing to maladaptive stress responses and depression pathogenesis. It is, however, still unclear exactly how peripheral circulating signals affect the neuronal circuits involved in mood regulation. Indeed, from a CNS perspective, the BBB form complex interactions with microglia, the resident immune cells of the brain, but also with astrocytes, pericytes and neurons as part of the neurovascular unit. Under pathological conditions, for example stroke or traumatic brain injury, biphasic opening of the BBB occurs highlighting the complexity of neurovascular responses to inflammation (Greene, Hanley & Campbell, 2019). In psychiatric research, transport through the BBB has been a challenge for several promising mood disorders‐related drugs in the past decades. An intriguing study from the Duman laboratory recently reported that brain‐derived neurotrophic factor (BDNF)‐mediated antidepressant effects requires vascular endothelial growth factor (VEGF) release, generally associated with endothelium inflammation, increased BBB permeability and formation of blood vessels (Van Dyken & Lacoste, 2018). This seems counterintuitive considering that loss of BBB integrity is associated with stress vulnerability and depression (Cheng et al., 2018; Menard et al., 2017; Pearson‐Leary et al., 2017). However, VEGF can be secreted by various cell types including neurons, astrocytes and endothelial cells and, in stroke, has deleterious pro‐inflammatory than beneficial angiogenic effects (Sandoval & Witt, 2008). Reciprocal interactions between VEGF and BDNF derived specifically from neurons appear to be necessary to improve depression‐like behaviours in male mice. Thus, the authors propose that both growth factors could be involved in the neurotrophic and antidepressant effects of ketamine, a promising fast acting drug for treatment‐resistant MDD (Deyama, Bang, Kato, Li & Duman, 2019). This paper highlights the importance of conducting cell‐specific manipulations to properly assess the contribution of neuronal, immune and vascular adaptations in mood disorders. Increasing availability of genetic mouse models and viral vectors dedicated to endothelial and glial cells is key to providing a better understanding of the neurovascular cellular and molecular mechanisms involved in stress responses. This is reinforced by several studies shedding light on astrocyte–neuron communication in mood disorders and antidepressant response (for review, see Sanacora & Banasr, 2013). Finally, most of the preclinical studies conducted in rodents have been performed in males soon after the last episode of stress and it will be interesting to evaluate whether chronic stress induces sex‐specific long‐lasting effects on the BBB, for example, through epigenetic changes.

## 5 CONCLUSIONS

In this review, we summarized the current knowledge regarding the mechanisms involved in the neurobiology of depression and resilience focusing on immune system modulation, vascular health and sex differences. MDD affects many people worldwide and is known to be the main risk factor for suicide (Angst et al., 1999), causing a big social and economic impact, strongly contributing to the years lived with incapacity (Vos, Barber, Bell, Bertozzi‐Villa & Biryukov, 2015). Depression is a biologically heterogeneous disease involving several systems; however, the pathophysiology of MDD is poorly understood and a better comprehension of the mechanisms underlying this mood disorder could drive the discovery of new therapeutic avenues for treating MDD patients or at least improving their quality of life. Activation of the HPA axis and ANS is frequently observed in MDD patients and mice subjected to chronic stress. In addition, increased attention has been given to the impact of the immune system and neurovascular health in depression (Chan, Cathomas & Russo, 2019; Hodes et al., 2015; Hodes et al., 2016; Ménard et al., 2017). Therefore, considering in parallel multiple physiological systems involved in stress responses and depression appears necessary and should be encouraged to shed light into new directions for treating this disorder in a non‐neuron‐centric way.

Chronic stress exposure is the most important environmental risk factor in the development of MDD (Straub et al., 2011), and some of the current rodent models used to study depression are based on this approach, reinforcing translational value. The CSDS model allows to not only study the negative impact of social stress but also coping behaviours, with one‐third of mice not developing depression‐like behaviour but evoking central and peripheral adaptive coping mechanisms against stress, remaining resilient (Golden, Covington, Berton, Russo & Russo, 2011). It is important to note that, after CSDS or other rodent models defining a resilient subgroup of individuals, resilient animals are not like unstressed controls and do present a specific set of biological alterations leading to stress resilience. Most of the studies have been focused only in the maladaptive changes during MDD and not on the mechanisms driving pro‐coping adaptations. Investigation of resilience biology is still new, and knowledge of cellular, molecular and epigenetic mechanisms involved is in its infancy. Nevertheless, this field is full of great promise and characterizing resilience‐associated endocrine, immune and vascular adaptations taking into account sex differences in the various stress paradigms and rodent models of depression will give future directions for therapeutic strategies aiming at promoting coping behaviour in MDD patients.

For too long sex differences have been neglected despite a higher prevalence of MDD in women (World Health Organisation, 2017). Important biological differences exist in response to stress, and women and men show a sex‐specific immune profile at basal level and during MDD pathology (Rainville et al., 2018). Additionally, many signalling pathways and circuits present sexually dimorphic expression after stressful experience in mice (Hodes et al., 2014) and in subjects diagnosed with MDD (Labonté et al., 2018). The lack of studies in female mice and in women might have contributed, at least partly, to an increased rate of resistance in women to currently available treatments. Studies evaluating sex differences will shed light on novel mechanisms involved in depression, hopefully improving treatment and reducing relapse rates.

## CONFLICT OF INTEREST

The authors have no conflict of interest to declare.

## AUTHOR CONTRIBUTIONS

KAD and LDA contributed equally to the manuscript. KAD and LDA wrote the manuscript with support of CM for outline and key papers. FNK made the figures and LDA the tables. FNK, ET, ML and CM edited and revised the manuscript. All authors commented, edited and revised the final version of the manuscript, figures and tables.
